# Reconciling Estimates of Cell Proliferation from Stable Isotope Labeling Experiments

**DOI:** 10.1371/journal.pcbi.1004355

**Published:** 2015-10-05

**Authors:** Raya Ahmed, Liset Westera, Julia Drylewicz, Marjet Elemans, Yan Zhang, Elizabeth Kelly, Rajko Reljic, Kiki Tesselaar, Rob J. de Boer, Derek C. Macallan, José A. M. Borghans, Becca Asquith

**Affiliations:** 1 Institute for Infection and Immunity, St. George’s, University of London, London, United Kingdom; 2 Laboratory of Translational Immunology, Department of Immunology, University Medical Center Utrecht, Utrecht, The Netherlands; 3 Theoretical Biology & Bioinformatics, Utrecht University, The Netherlands; 4 Department of Medicine, Imperial College, London, United Kingdom; Emory University, UNITED STATES

## Abstract

Stable isotope labeling is the state of the art technique for *in vivo* quantification of lymphocyte kinetics in humans. It has been central to a number of seminal studies, particularly in the context of HIV-1 and leukemia. However, there is a significant discrepancy between lymphocyte proliferation rates estimated in different studies. Notably, deuterated ^2^H_2_-glucose (D_2_-glucose) labeling studies consistently yield higher estimates of proliferation than deuterated water (D_2_O) labeling studies. This hampers our understanding of immune function and undermines our confidence in this important technique. Whether these differences are caused by fundamental biochemical differences between the two compounds and/or by methodological differences in the studies is unknown. D_2_-glucose and D_2_O labeling experiments have never been performed by the same group under the same experimental conditions; consequently a direct comparison of these two techniques has not been possible. We sought to address this problem. We performed both *in vitro* and murine *in vivo* labeling experiments using identical protocols with both D_2_-glucose and D_2_O. This showed that intrinsic differences between the two compounds do not cause differences in the proliferation rate estimates, but that estimates made using D_2_-glucose *in vivo* were susceptible to difficulties in normalization due to highly variable blood glucose enrichment. Analysis of three published human studies made using D_2_-glucose and D_2_O confirmed this problem, particularly in the case of short term D_2_-glucose labeling. Correcting for these inaccuracies in normalization decreased proliferation rate estimates made using D_2_-glucose and slightly increased estimates made using D_2_O; thus bringing the estimates from the two methods significantly closer and highlighting the importance of reliable normalization when using this technique.

## Author Summary

Stable isotope labeling is used to quantify the rate at which living cells proliferate and die in humans. It has been central to a number of seminal studies, particularly in viral infections such as HIV-1, and leukemia. However, different labels (deuterated water or deuterated glucose) yield different estimates for the rate of cell proliferation and loss; this hampers our understanding and weakens our confidence in this important technique. We performed *in vitro* and *in vivo* experiments as well as a new analysis of existing data to directly compare the two labels. This reveals that a major source of the discrepancy lies in the difficulty of assessing deuterated glucose availability. We reconcile published studies and provide recommendations to avoid this problem in the future.

## Introduction

Quantification of lymphocyte kinetics is vital for our understanding of immune cell dynamics in health and disease. The development [1,2] of stable isotope labeling techniques, using either deuterium labeled glucose (D_2_-glucose) or deuterium labeled water (D_2_O), has enabled the safe quantification of lymphocyte turnover in humans *in vivo*. This has provided unprecedented insight into immunity in healthy individuals, as well as in various conditions, including aging, viral infection, diabetes and leukemia [3–14].

Despite this breakthrough, estimates of lymphocyte kinetics (i.e. proliferation and loss) are known to differ up to 10-fold between stable isotope labeling studies, with D_2_-glucose labeling consistently yielding higher proliferation and loss rates than D_2_O labeling [6,8,15–17]. We have previously shown that short labeling periods can yield higher estimates of proliferation and loss than long labeling periods [18–20]. In the case of proliferation rate estimates this is because, with long labeling periods, the label in rapidly turning over cell subpopulations can saturate leading to an underestimate of the proliferation rate [18,19]. In the case of loss, this is because the loss rate estimated pertains only to the labeled cell population and the composition of the labeled cell population will change as the duration of label administration changes [20], with a stronger bias towards rapidly dividing cells when labelling periods are shorter. Since D_2_-glucose labeling protocols usually involve a much shorter labeling period than D_2_O labeling protocols, the relationship between the duration of label administration and the estimate of proliferation and loss explains some of the difference between kinetics obtained using the two techniques. However, even after correcting for the length of the labeling period, our analysis here shows that significant discrepancies remain. This raises the concern that there may be fundamental differences between D_2_O labeling and D_2_-glucose labeling that affect the estimates of lymphocyte kinetics they produce.

D_2_-glucose and D_2_O labeling experiments differ in several aspects, both in the exact experimental procedures as well as in the way the experimental data are translated into the biological parameters of interest. D_2_-glucose is typically administered for short periods of time (hours or days). The pool of blood glucose is small and has a high rate of turnover, so rapid up- and de-labeling can be achieved [15,16]. Although any part of the glucose molecule could be labeled, most studies have used 6,6-D_2_-glucose. During *de novo* purine and pyrimidine synthesis, the two non-exchangeable deuteriums on the 6-position are carried over into the 5-position carbon of the pentose moiety of DNA precursors (as C1 is lost and C6 is redesignated C5) [2]. Deuterium enrichment is measured (by mass spectrometry) in the pentose moiety of the DNA of the cell population of interest. When D_2_-glucose labeling experiments are conducted *in vitro*, enrichment levels in DNA never reach media enrichments but plateau at about 60–75% [2]. This has been attributed to intracellular dilution of labeled nucleotide triphosphates (NTP) by pre-formed NTP, by other pentose precursors, and by unlabeled salvage pathway synthesis. Since the short labeling periods associated with D_2_-glucose preclude the use of a completely replaced population to derive product-based estimates of *in vivo* precursor enrichments, these plateau tissue culture values have been used to correct for (an assumed similar level of) intracellular dilution *in vivo*, by applying a scaling factor in the range 0.6–0.75 [19]. Within this manuscript we refer to this intracellular dilution factor as *b*_*g*_. In addition to adjusting for intracellular dilution, it is necessary to correct for the label availability, which is estimated by measuring the label enrichment in glucose in blood plasma at multiple time points.

By contrast D_2_O is usually administered for several weeks. Intermediary metabolism introduces the deuterium in place of hydrogen in *de novo* synthesized deoxyribose. To determine the level of incorporation, mass spectrometric analysis is performed on the deoxyribose moiety of purine nucleotides [1]. The observed deuterium incorporation in the DNA from the cell population(s) of interest is normalized to the maximal level of deuterium incorporation that deoxyribose can attain, which is typically determined in the same individual in a population with rapid turnover, such as granulocytes, monocytes or thymocytes [8]. This maximum enrichment attainable is determined by a scaling factor and the level of D_2_O in the body. The scaling factor, which is analogous to the intracellular dilution factor *b*_*g*_ for D_2_-glucose, has been variously referred to as the amplification factor or *c* [8]. Here, to emphasize analogy to *b*_*g*_, we refer to it as *b*_*w*_. Deoxyribose contains seven non-exchangable hydrogen atoms, any of which might potentially be replaced by deuterium [1,21]. Consequently, the maximum level of enrichment seen in deoxyribose exceeds that seen in plasma and the scaling factor, *b*_*w*_, is greater than 1; typically *b*_*w*_ is in the range 3.5–5.2 [8]. Body water turns over relatively slowly, so enrichment in the body fluids reaches its maximum and is washed-out from the body more slowly than D_2_-glucose. Consequently, there is still considerable *de novo* DNA labeling long after the label has been withdrawn. A correction for the level of D_2_O present in the body fluids is made by taking the enrichment of blood plasma or urine samples into account [8].

In addition to the methodological differences between D_2_O and D_2_-glucose labeling protocols there are differences in the way the compounds are synthesized into deoxyribose [1,19], in the distribution of water and glucose throughout the body, in the transport of water and glucose into cells, and in the feedback mechanisms that control water and glucose levels. Potentially, some or all of these differences could cause the compounds to give different estimates of cell kinetics. To date a direct comparison of the two techniques has not been performed. We sought to address this problem.

We performed stable isotope labeling experiments *in vitro* and in mice using D_2_-glucose and D_2_O, while keeping every other aspect of the study identical. This showed that, for T cells *in vitro* and murine splenocytes and PBMC *in vivo*, biochemical differences between the compounds do not lead to differences in parameter estimates. Instead, we found that proliferation rate estimates made using D_2_-glucose in mice were susceptible to difficulty in estimating rapidly fluctuating blood glucose enrichment levels. We therefore hypothesized that the measurement of deuterium enrichment in blood glucose may be an unreliable estimate of the precursor enrichment. This hypothesis was supported by a new analysis of two published D_2_-glucose studies [6,13]. Analogous problems, albeit of a much smaller magnitude, were also found in a published D_2_O study [8]. We show that adjusting for these presumed inaccuracies in normalization decreases the proliferation rates estimated in the D_2_-glucose studies whilst simultaneously increasing the proliferation rates from the D_2_O labeling study, thus bringing these estimates significantly closer.

## Results

### Discrepancy between proliferation rate estimates obtained using D_2_O and D_2_-glucose in humans despite adjusting for the length of the labeling period

**Fig 1:**
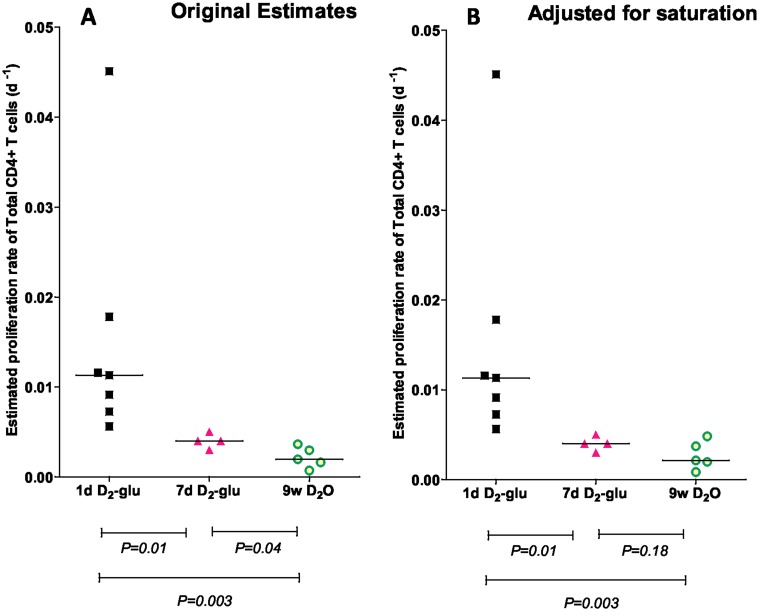
Total CD4^+^ T cell proliferation rates estimated from D_2_-glucose and D_2_O labeling studies in humans. Each symbol represents a different individual, horizontal lines represent the median of the estimates. **(A)** Proliferation rates estimated using the kinetic heterogeneity model. **(B)** Proliferation rates estimated after adjusting for saturation (using the multi-exponential model for the nine-week (9w) D_2_O and seven-day (7d) D_2_-glucose labeling, and the kinetic heterogeneity model for one-day (1d) D_2_-glucose labeling). Although correcting for the saturation of rapidly turning over subpopulations helps to bring the estimates closer (by increasing the proliferation rate estimates obtained in the nine-week D_2_O labeling study) significant discrepancies remain.

Long labeling periods can lead to an underestimate of cell proliferation rates due to saturation of label [18]. We therefore investigated whether adjusting for the length of the labeling period resolves the discrepancy between estimates of lymphocyte proliferation obtained using D_2_-glucose and D_2_O labeling. We focused on three published studies in healthy humans where detailed data were available: a 9 week D_2_O labeling study [8], a seven-day D_2_-glucose labeling study [13] and a one-day D_2_-glucose labeling study [6] and compared the labeling of total CD4^+^ T lymphocytes (Methods). We analyzed the data from the seven-day D_2_-glucose and the 9 week D_2_O labeling study using a multi-exponential model [22]. The multi-exponential model (Methods) consists of *N* homogeneous subpopulations; proliferation and death within each subpopulation is random and occurs at a constant rate and each subpopulation is assumed to be independently at equilibrium (i.e. proliferation = death). The multi-exponential model allows for saturation of label in rapidly turning over subpopulations and effectively adjusts for the length of the labeling period. There were fewer data points available for the one-day D_2_-glucose study and so the multi-exponential model could not be used (since it has a relatively large number of free parameters). However, saturation is unlikely to be an issue with such a short labeling period so we used the alternative, kinetic heterogeneity model [6,20]. The kinetic heterogeneity model is a model that deals with heterogeneity implicitly by postulating that labeled cells may not be representative of the whole population and thus the disappearance rate of labeled cells (d*) may not be equal to the proliferation rate of the whole population (*p*) even for populations at equilibrium (Methods). The advantage of the kinetic heterogeneity model over the multi-exponential model is that it has fewer parameters (2 compared with 2*N*-1 for the multi-exponential model) and, in the absence of saturation, yields identical estimates of turnover. We confirmed that, even for the seven-day D_2_-glucose study, the multi-exponential and kinetic heterogeneity models give identical proliferation rate estimates, suggesting that, at least for CD4^+^ T cells, saturation is only a problem with the long labeling periods associated with D_2_O. The resulting proliferation rate estimates are shown in Fig 1. The differences between the one-day and seven-day D_2_-glucose estimates and between the one-day D_2_-glucose and 9 week D_2_O labeling estimates were significant (*P* = 0.01 and *P* = 0.003 respectively, two-tailed Mann-Whitney). Thus, although correcting for the saturation of label in rapidly turning over subpopulations helps to reduce the difference between the estimates (by increasing the proliferation rate estimates obtained in the 9 week D_2_O labeling study) significant discrepancies remain. Strikingly, these discrepancies are not only apparent in the comparison of D_2_-glucose and D_2_O labeling studies but also between the different D_2_-glucose labeling studies. That this is not an artefact of the modeling can be seen by studying the normalized experimental data. In the one-day D_2_-glucose study, labeling for only one-day resulted in deuterium enrichment in CD4^+^ T cells at day 3–5 similar to the enrichment obtained after 4 days of labeling in the seven-day labeling study (S1 Fig). This basic difference in data underlies the difference in estimated proliferation rates.

### In vitro, no fundamental differences between D_2_-glucose and D_2_O labeling

**Fig 2:**
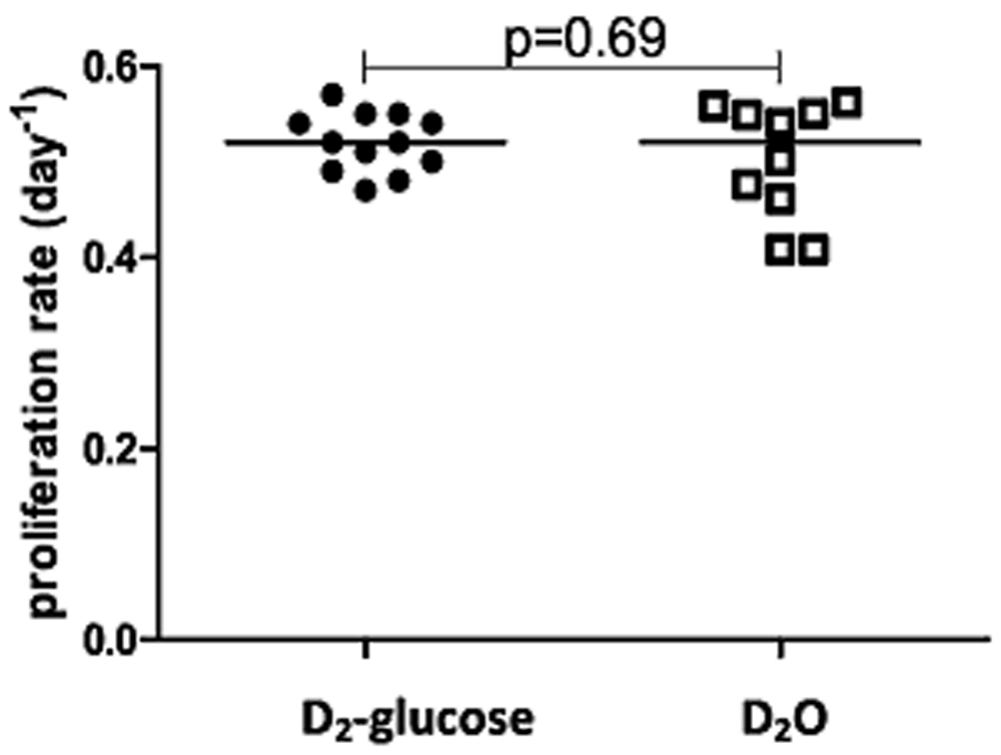
Proliferation rates of Jurkat cells growing *in vitro* estimated using D_2_-glucose and D_2_O labeling. Each symbol represents the proliferation rate estimated in a separate experiment, horizontal lines represent the median of the estimated proliferation rates.

We sought to directly compare D_2_O and D_2_-glucose by labeling an immortalized human T cell line (Jurkat) *in vitro* either with D_2_O or with D_2_-glucose or with both compounds simultaneously. In all cases cells were labeled for seven-days with a seven-day wash-out phase. Data were normalized using the conventional strategy. Specifically, we adjusted for the level of label availability in both the D_2_-glucose and the D_2_O labeling experiments based on measured deuterium levels in culture media, and we estimated the maximum level of label enrichment that could be attained by fitting *b*_*w*_ in the case of D_2_O and using a fixed value of *b*_*g*_ = 0.65, based on the maximal end-product enrichment of such cells *in vitro*, in the case of D_2_-glucose labeling. Existing mathematical models, which assume lymphocyte steady state, were adjusted to allow for a growing cell population (Methods) and fitted to the normalized data. We assumed cell death in the cultures was negligible. We found that the estimates of proliferation obtained using D_2_O (*p* = 0.50±0.06 d^-1^, *b*_*w*_ = 3.46±0.27) were similar to those obtained using D_2_-glucose (*p* = 0.52±0.03 d^-1^), and the difference between the estimates was not significant (*P* = 0.69, two-tailed Mann-Whitney), Fig 2, S1 Table, and S2 Fig. We conclude that estimates from *in vitro* D_2_-glucose and D_2_O labeling are in good agreement and that biochemical differences between D_2_O and D_2_-glucose did not lead to differences in proliferation rate estimates in this experiment.

### D_2_-glucose and D_2_O labeling experiments in mice: sensitivity to blood glucose measurements

To compare D_2_-glucose and D_2_O labeling *in vivo* we conducted a seven-day oral labeling study of mice. For one group of mice both the feed and the drinking water were spiked with D_2_O to an enrichment of 8%; the other group received feed spiked with D_2_-glucose (comprising ~30% of glucose intake). Identical chemical composition of feed between groups was maintained by spiking the feed for D_2_O-labeled mice with “unlabeled” (normal) glucose and for the glucose-labeled mice with “unlabeled” water. Food consumption was similar between the groups and all mice continued to gain weight. Deuterium enrichment was measured in blood plasma, PBMC, splenocytes, and thymocytes.

**Fig 3:**
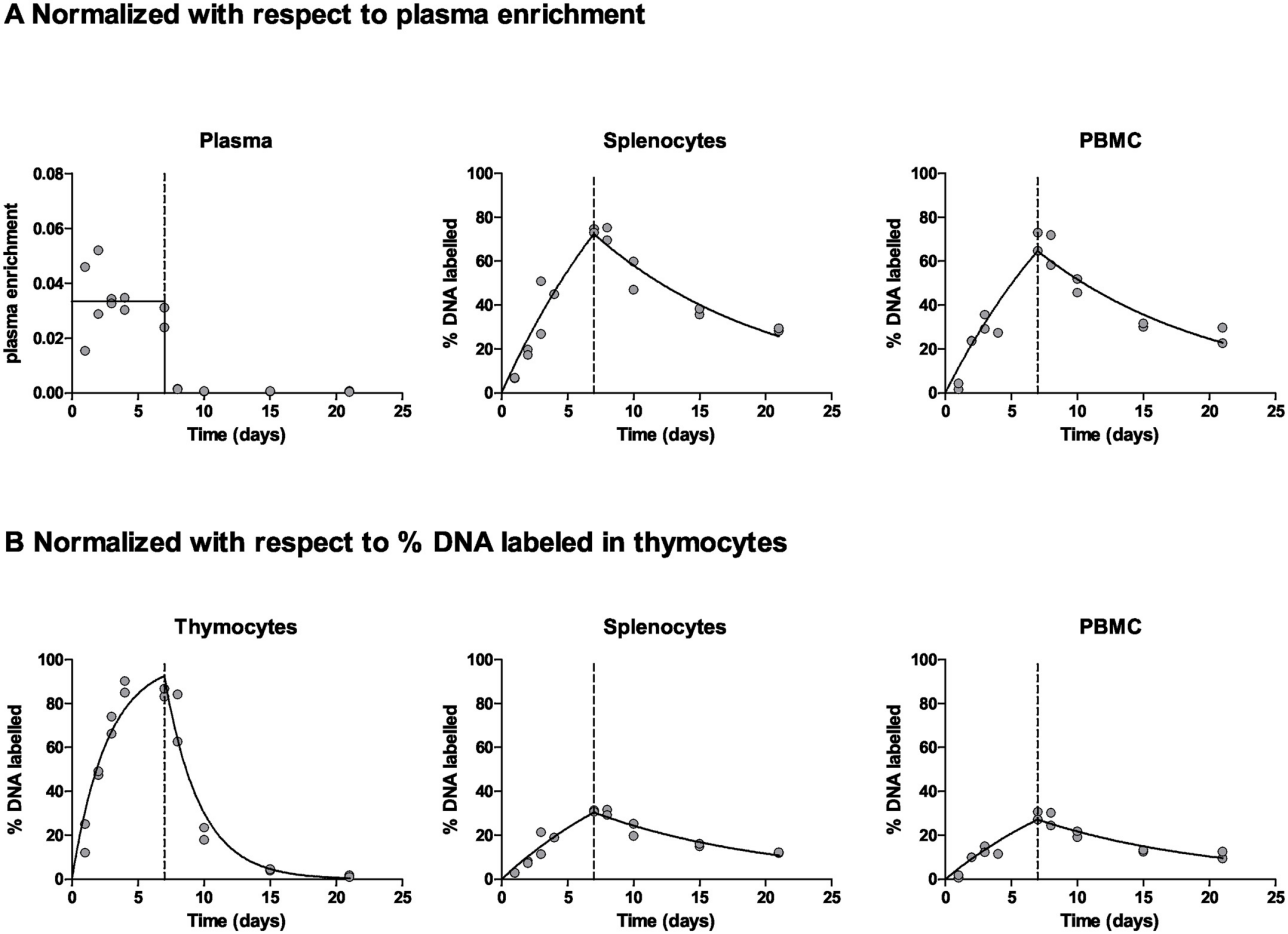
Enrichment curves of D_2_-glucose labeled mice. Best fits to the fraction of deuterium enrichment in plasma, and the percentage of label enrichment in the DNA of PBMC, splenocytes and thymocytes after seven-days of D_2_-glucose labeling. Dots represent individual mice. The end of label administration at day 7 is marked by a dashed vertical line. **(A) Conventional normalization:** measurements are normalized to the mean plasma enrichment x *b*_*g*_. **(B) Normalized with respect to thymocytes**: thymocytes were used to determine the maximum percentage of labeled DNA that cells could possibly attain (Methods), and all measured enrichments were scaled to this maximum. The raw data of the % DNA labeled in splenocytes and PBMC is the same in panel A and B but has been normalized using two different approaches.

**Fig 4:**
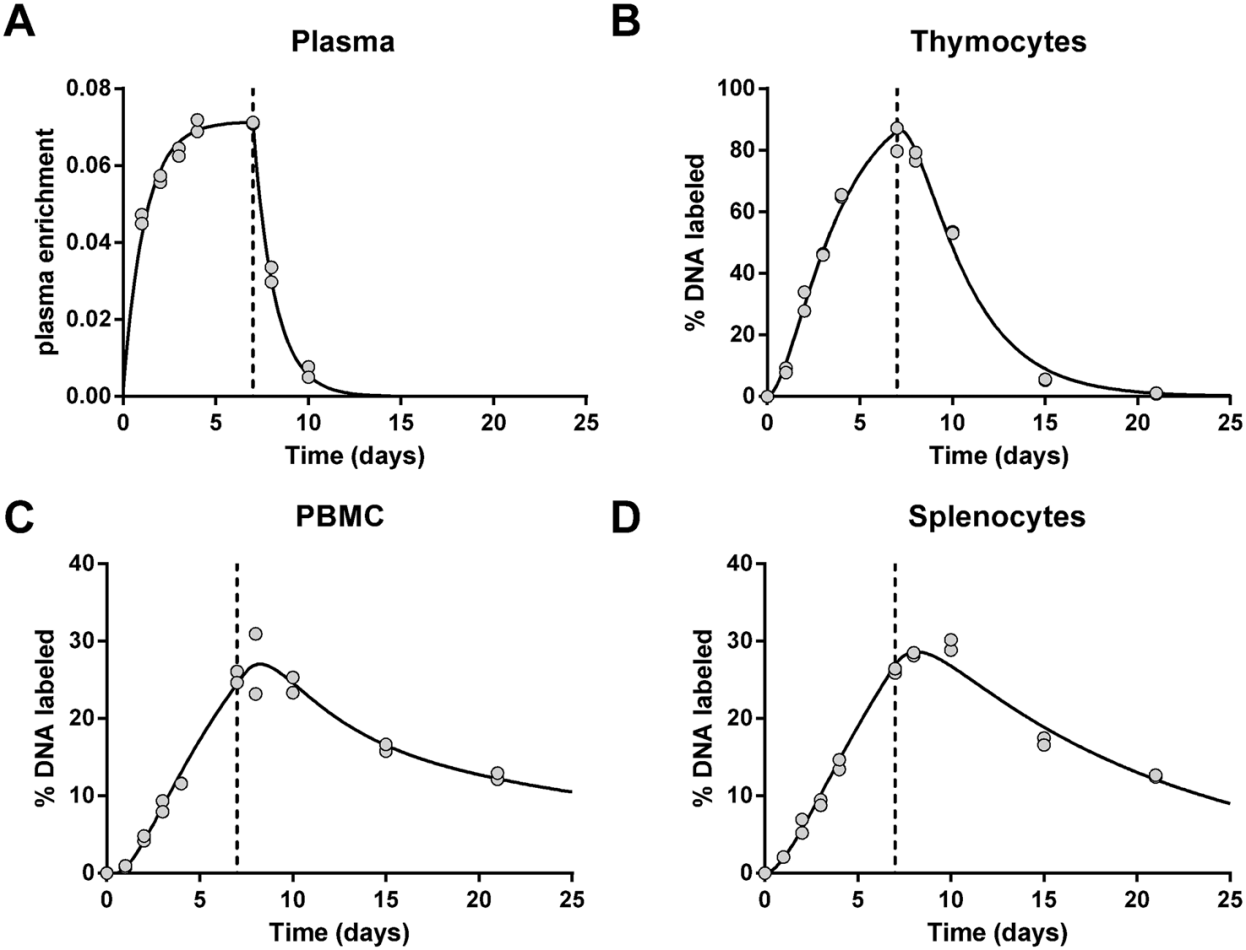
Enrichment curves of D_2_O labeled mice. Best fits to the fraction of deuterium enrichment in plasma **(A)** and the percentage of label enrichment in the DNA of thymocytes **(B)**, PBMC **(C)**, and splenocytes **(D)** after seven-days of D_2_O labeling. Thymocytes were used to determine the maximum percentage of labeled DNA that cells could possibly attain (Methods), and all measured enrichments were scaled to this maximum. Dots represent individual mice. The end of label administration at day 7 is marked by a dashed vertical line.

**Fig 5:**
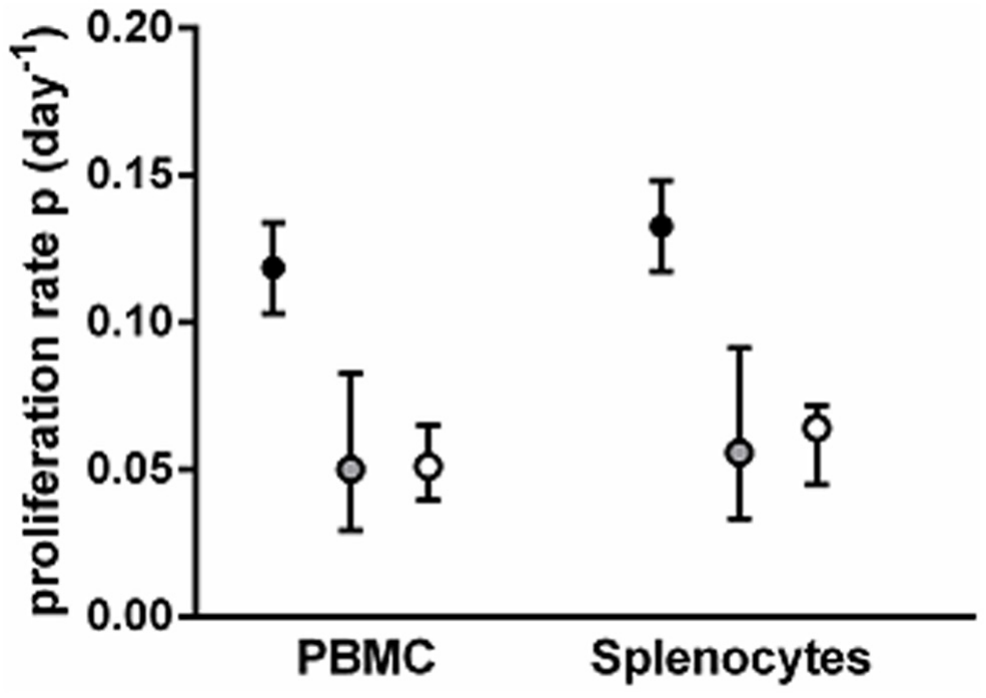
Proliferation rate estimates from 1 week D_2_-glucose and D_2_O labeling in mice. The average proliferation rate of PBMC and splenocytes, obtained by fitting the kinetic heterogenity model to the data collected during labeling with D_2_-glucose with conventional normalization (using 0.65 × plasma AUC, filled circles), labeling with D_2_-glucose with normalization to thymocytes (grey-filled circles) and labeling with D_2_O with conventional normalization i.e. to thymocytes (open circles). Bars represent 95% confidence intervals; AUC = area under the curve.

All cell types showed a progressive increase in DNA enrichment. Strikingly, the maximum enrichment in thymocyte DNA in glucose-labeled mice (about 4.5%) exceeded the estimated precursor (D_2_-glucose) enrichment, which averaged about 3.3% during the labeling period. Initially, the raw data were normalized following the conventional approach. That is, both D_2_-glucose and D_2_O data were first adjusted for plasma deuterium enrichment. The data were then scaled to the maximal level of enrichment; for D_2_O labeling this was determined using the plateau enrichment of a rapidly turning over cell population (thymocytes in this case), and for D_2_-glucose by using the *in vitro* derived constant factor *b*_*g*_ = 0.65. The kinetic heterogeneity model was then fitted to the normalized data. This analysis (Figs 3A and 4) yielded substantially different proliferation rates for the D_2_O and D_2_-glucose labeling experiments (Fig 5). Similar (differences in) estimates were found using a multi-exponential model.

**Fig 6:**
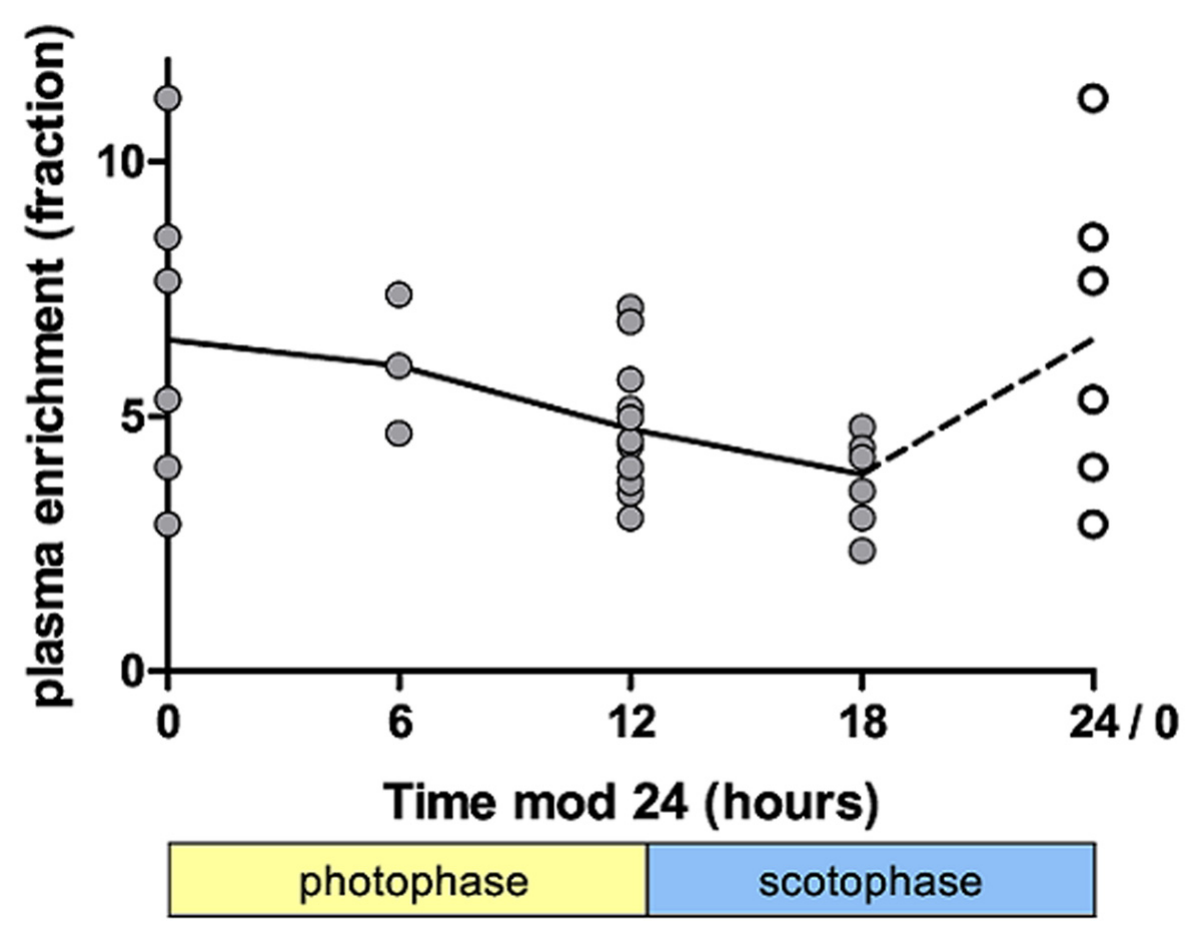
Impact of light-dark cycle on plasma glucose enrichment in mice. The fraction of deuterium enrichment of plasma glucose is plotted against time since the start of label modulo 24 h. Data from mice housed under reversed day/night condition is translated by 12h so that for all mice 0h represents the start of light phase (8am for normal conditions, 8pm for reversed day/night conditions). Grey circles represent measurements from individual mice, the line connects the medians, the points plotted at 24h/0h are duplicates of the 0h data.

We were concerned by the large variations in D_2_-glucose plasma enrichment (Fig 3A) and the potential impact of mouse diurnal feeding patterns. By contrast, D_2_O plasma enrichment showed very little data variability (Fig 4A). In order to improve our estimate of glucose enrichment and reduce sampling error we repeated the D_2_-glucose arm of the experiment under identical conditions but with more frequent blood sampling (n = 27 plasma samples in 12 mice over seven-days) including both day and night sampling (facilitated by a reverse day-night cycle). As expected, enrichment in DNA from cells (thymocytes, splenocytes, PBMC) reached very similar values to those from the first experiment; specifically thymus labeling reached a maximum of about 5.1% with a modeled plateau of 4.9%. As before, large variations in plasma glucose enrichment were seen (S3 Fig) although these tended to stabilize with time, albeit at lower levels than in the first 24 hours, suggesting that label may be handled differently in the early stages of the experiment compared to later. When light-dark patterns were analyzed, a pattern could be discerned in which labeling reached a minimum in the middle of scotophase in line with previous publications [23] (Fig 6). Importantly, we found similar evidence for a diurnal pattern in humans that were labeled with D_2_-glucose [6]. In this study, subjects received D_2_-glucose by continuous infusion for 24h but also ate unlabeled low–carbohydrate meals. The plasma enrichment in these individuals tended to increase during the night (S4 Fig), which is to be expected as the intake of deuterated glucose remained constant whilst the intake of unlabeled glucose ceased as the individual sleeps (subjects were not woken to receive nutrition during the night).

We reasoned that variability in the plasma enrichment may make estimation of the D_2_-glucose availability problematic, we therefore reanalyzed the D_2_-glucose experiment normalizing to the estimated plateau enrichment of thymocytes, rather than using the mean plasma enrichment x *b*_*g*_. This reanalysis (Fig 3B) caused a dramatic reduction in proliferation rate estimates from the D_2_-glucose experiment resulting in good agreement between the proliferation rate estimates from the D_2_-glucose and D_2_O labeling experiments. The average proliferation rate of PBMC was 0.050 d^-1^ with D_2_-glucose and 0.051 d^-1^ with D_2_O; the average proliferation rate of splenocytes was 0.056 d^-1^ with D_2_-glucose and 0.064 d^-1^ with D_2_O (Fig 5). Again, similar estimates were found using a multi-exponential model. In short, when the D_2_-glucose and D_2_O data were analyzed using an identical normalization procedure, the estimates of cell proliferation were in good agreement; however when conventional normalization approaches (i.e. normalizing to the plasma deuterated glucose level) were used, differences emerged. These findings suggest that biochemical differences between these deuterium labeled compounds do not influence lymphocyte proliferation estimates in mice. However, the difficulty of estimating label availability throughout the course of the experiment may cause discrepancies.

### Reanalysis of one-day and 1 week D_2_-glucose labeling experiments in humans

**Fig 7:**
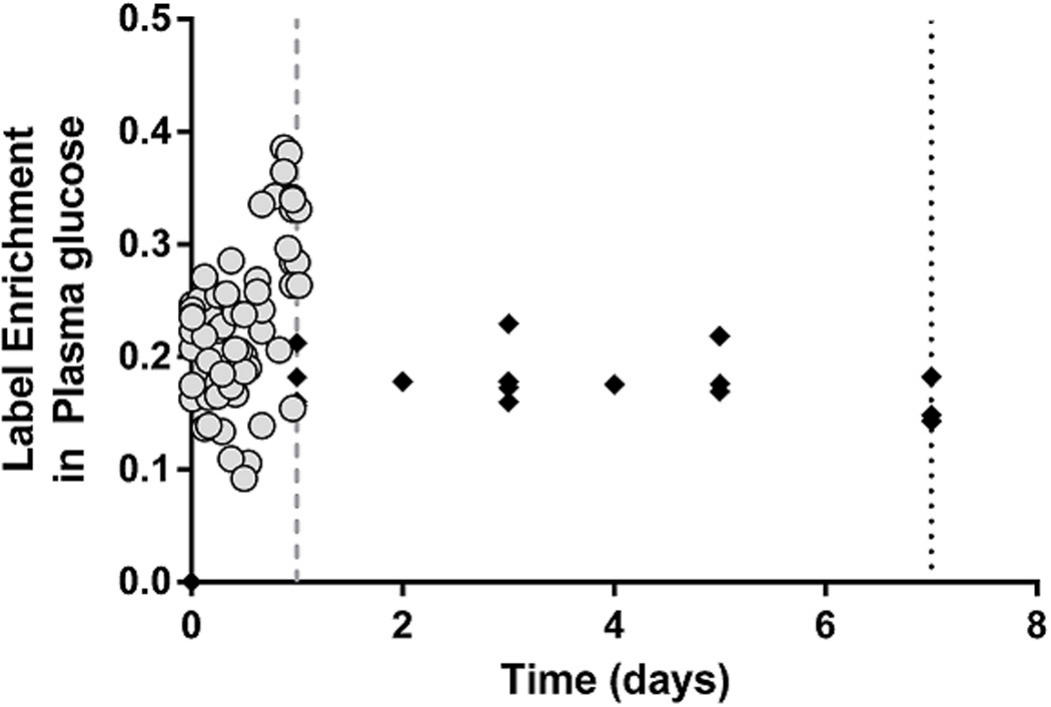
Deuterium enrichment in plasma glucose in one-day and seven-day D_2_-glucose labeling studies of humans. The measured enrichment in plasma glucose in the one-day (grey circles) and seven-day (black diamonds) D_2_-glucose labeling studies. The end of the one-day and seven-day labeling periods are marked by a dashed grey line and a dotted black line respectively. Although subjects in the one-day labeling experiment (N = 8) received approximately twice as much deuterated glucose per day as subjects in the seven-day labeling study (N = 4), their median label enrichment is only slightly higher. Median plasma glucose enrichment in the one-day labeling study = 23%, median plasma glucose enrichment in the seven-day labeling study = 17%.

Based on these *in vitro* and *in vivo* studies we hypothesized that normalization using the observed D_2_-glucose enrichment in the blood is problematic as this quantity fluctuates markedly and shows systematic diurnal variation, making it difficult to assess the label enrichment over the course of the study. To test this hypothesis we analyzed two published D_2_-glucose labeling studies (one-day and seven-day D_2_-glucose labeling of healthy individuals [6,13] where labeling in a rapidly turning over population (monocytes) was also available. If the average plasma enrichment during the labeling period is a good measure of intracellular DNA precursor enrichment, then, if we estimate the plateau enrichment of monocytes (i.e. the maximum label they can attain) this should be 100%. However if, as we hypothesize, the average D_2_-glucose enrichment in plasma during the labeling period is a poor surrogate for precursor enrichment, then the plateau enrichment of the monocytes will be significantly different from 100%. We constructed a model of monocyte development in which monocyte progenitors in the bone marrow proliferate, mature, exit into the blood and from there migrate into tissue to differentiate into macrophages or dendritic cells (Methods). We fitted this model to the normalized monocyte data from one-day and seven-day D_2_-glucose labeling studies and estimated the plateau enrichment of monocytes (Table 1 and S5 Fig). This analysis showed that, for the one-day labeling study, subjects tended to have a plateau enrichment significantly above 100% whereas for the seven-day labeling study, mean plateau enrichment was consistent with or slightly lower than 100% (Table 1). Repeating this analysis with a simpler but less physiological model confirmed these results (“delayed observation” model, Methods). For every model, the plateau enrichment was significantly higher in the one-day than in the seven-day labeling study (*P* = 0.048, *P* = 0.012, *P* = 0.012 for the bone marrow, delayed observation and a weighted combination of both models respectively, two-tailed Mann-Whitney). We conclude that there is evidence that the mean plasma enrichment may have underestimated the label available to dividing cells during the one-day D_2_-glucose study (and potentially overestimated label availability during the seven-day study). This conclusion is supported by an analysis of the deuterium enrichment in blood plasma glucose in the two studies. If we compare the labeling protocol of the one-day and seven-day D_2_-glucose studies, we see that the subjects in the one-day labeling study were infused with approximately twice as much D_2_-glucose per day as subjects in the seven-day labeling study. Despite this, the median plasma enrichment measured was only slightly higher in the one-day labeling study (Fig 7). These findings are consistent with the hypothesis either that the measured plasma enrichment in the one-day labeling study may have underestimated label availability (which would in turn have led to an overestimation of the T cell proliferation rate), or that carbohydrate intake, and thus glucose flux, was higher in subjects in the one-day study.

**Table 1:** Estimates of the plateau enrichment of monocytes in humans.

		Plateau enrichment of monocytes (%)		
		BM	Delayed obs.	weighted
1-day D_2_-glucose	**C02**	98±0	188±0	143±32
	**C03**	110±1	211±0	161±36
	**C04**	100±1	192±0	146±32
	**C05**	109±1	208±0	159±35
	**C06**	144±1	277±0	211±47
	**C07**	131±1	251±0	191±42
	**C08**	146±1	280±0	213±47
	**C10**	153±1	293±0	223±50
	**MEAN**	**124±22**	**237±43**	**181±32**
7-day D_2_-glucose	**C2**	90±4	105±3	97±6
	**C3**	102±4	120±3	111±7
	**C4**	79±4	92±3	85±5
	**MEAN**	**90±12**	**106±14**	**98±13**

The maximum level of label that monocytes can attain (expressed as a percentage) was estimated for a one-day D_2_-glucose labeling study (subjects C02-C10) and a seven-day D_2_-glucose labeling study (subjects C2–C4). Two different models were considered: the “BM”(bone marrow) model and the “delayed obs.” (delayed observation) model (Methods). We also calculated the average estimate of the plateau by weighing each fit with the corresponding Akaike weight [24]; these estimates are given in the column “weighted”. Estimates are shown ± standard deviation (sd). The sd in the “weighted” column reflects model uncertainty (whereas sd for the specified models assumes the model is correct). For every model the plateau enrichment was significantly higher in the one-day labeling study than in the seven-day labeling study (P = 0.048, P = 0.012, P = 0.012 for the BM, delayed obs. and weighted model respectively, two-tailed Mann-Whitney). In the one-day labeling study virtually all the plateau estimates were higher than 100% (23/24 ≥ 100%, 1/24 <100%); for the seven-day labeling study estimates were evenly distributed either side of 100% (4/9 ≥ 100%, 5/9 < 100%).

### Normalization in D_2_O labeling studies in humans

These investigations, which reveal a problem with the normalization in D_2_-glucose labeling studies, prompted us to also examine the normalization in D_2_O studies. Again, we focused on the 9 week D_2_O labeling study [8] where detailed data were available. In the published study the data in all T cell subsets were normalized to the maximum enrichment in granulocytes. Maximum enrichment in granulocytes was estimated based on the measured enrichment in urine and on an estimate of the intracellular scaling factor *b*_*w*_ (*c* in the notation of the reporting paper [8]). The factor *b*_*w*_ was estimated using a simple model in which granulocytes in the body were assumed to be a homogeneous population with a single rate of proliferation which was assumed to be equal to a single death rate. This yielded estimates of the intracellular dilution *b*_*w*_ in the range *b*_*w*_ = 3.78–5.15. Since the label in granulocytes comes close to reaching a maximum, there is little scope for error in this plateau estimation. Nevertheless the model used is unphysiological. We therefore refitted the data using the more physiological “bone marrow” model we developed (Methods), in which granulocyte precursors proliferate in the bone marrow, followed by a lag period before they enter the blood and then exit the circulation. This model fitted the data well (S6 Fig) and in most cases resulted in an AICc (a measure of goodness of fit penalized by number of parameters [24]) considerably lower than the published model despite its increased complexity (the published model consistently overestimated the fraction of labeled cells during the delabeling phase). The estimates of *b*_*w*_ from the more physiological bone marrow model were significantly lower than the published estimates (*P = 0*.*01*, two-tailed paired Mann-Whitney, S7 Fig), though the effect is numerically small. This presumed overestimate of *b*_*w*_ in the published study will have caused an underestimate of the proliferation rate in all subpopulations. Correcting for this underestimate leads to a small proportional increase in T cell proliferation rate estimates of between 1%-10% (*P =* 0.0003, two-tailed paired Mann-Whitney).

## Discussion

Several independent lines of evidence indicate that the discrepancy between proliferation rates estimated with D_2_O and D_2_-glucose, once saturation of highly dynamic pools has been accounted for [18,19], is not explained by a fundamental difference between the compounds but instead can be explained in part by the difficulty of using deuterium enrichment in plasma glucose to quantify label availability. Firstly, we find no evidence, either *in vitro* or *in vivo*, for a biochemical difference between D_2_-glucose and D_2_O that impacts on cell proliferation rate estimates. Secondly, in mice *in vivo*, estimates of cell proliferation rates obtained using D_2_-glucose and D_2_O labeling agree if labeled precursor availability during the course of the study is estimated using a rapidly turning over population but not if it is estimated using plasma glucose enrichments. Thirdly, in humans there is evidence that deuterium enrichment in plasma glucose fluctuates markedly with a predictable increase in enrichment during the night. If the sampling strategy is unbalanced between day and night (or more specifically between post-prandial and fasting) then the plasma glucose measurements may not reflect label availability throughout the labeling period. In the case of the one-day labeling study, plasma glucose profiles were based on 6–8 readings during the labeling period but sampling during the night was avoided to reduce inconvenience to participants and as a result plasma glucose enrichment during the whole labeling period may be underestimated. Fourthly, if we compare the labeling protocol of the one-day and seven-day D_2_-glucose studies we see that the subjects in the one-day labeling study were infused with approximately twice as much D_2_-glucose per day as subjects in the seven-day labeling study. Despite this, the median plasma enrichment measured in the two experiments did not differ two-fold, again indicating a potential problem in assessing precursor availability. Finally, when we estimate the plateau enrichment of monocytes in the one-day and seven-day human D_2_-glucose labeling studies we find that, although the data have been normalized such that the maximum label incorporation should be 100%, there is evidence in the one-day labeling study that the maximum incorporation exceeds 100%. Again, this would suggest that the plasma glucose enrichment levels used to normalize the data in the one-day D_2_-glucose study underestimated the label availability.

If, as the data summarized above suggest, the label availability has been underestimated in the one-day human D_2_-glucose studies then a direct consequence will be that T cell proliferation rates will have been overestimated. Estimation of the magnitude of these errors is nontrivial. The difficulty of estimating the plateau enrichment in monocytes after such a short labeling period means that correction factors for each individual may be unreliable. Instead we focus on correction factors averaged across the cohort (Table 1). This indicates proliferation rates in the one-day study have been overestimated by a factor of 1.2–2.4 (16–57%). In contrast, we find little clear evidence for a problem with the normalization in the seven-day D_2_-glucose study; if anything there is perhaps a suggestion that label availability may have been overestimated and thus T cell proliferation underestimated (Table 1). In the case of the 9 week D_2_O study there is evidence again of a (numerically small) problem in the normalization. Reanalysis of existing 9 week D_2_O data with a new model indicates that both naive and memory CD4^+^ and CD8^+^ T cell proliferation rates may have been systematically underestimated by 1–10%. Reducing the T cell proliferation rate estimates from the one-day D_2_-glucose study and increasing the proliferation rates from the 9 week D_2_O study will bring the estimates from the two methods significantly closer.

There are at least three potential causes for an underestimate of label availability in the one-day D_2_-glucose study. Firstly, in the one-day labeling study the sampling of enrichment in plasma glucose was biased towards daytime (5–7 points during the day, only 1 fasted overnight point, S3 Fig) which may have led to a systematic underestimate of label availability as we have shown that enrichment increases during the night. In contrast, in the seven-day labeling study, all of the plasma enrichments were measured after overnight fasting (when plasma enrichment will be at its peak); thus in the seven-day study the average label availability may be overestimated resulting in an underestimate of the proliferation rate (consistent with the average plateau enrichment in monocytes of less than 100%, Table 1). In the case of the seven-day labeling study, this error may have been relatively small as carbohydrate intake was severely restricted thus reducing fluctuations due to dietary glucose. Secondly, the blood glucose pool is a small pool with a rapid flux, which is highly dependent upon dietary intake. Although glucose concentrations are very tightly regulated these regulation mechanisms are unlikely to distinguish between labeled and unlabeled glucose. Thus whilst total glucose levels are stable, the fraction of enrichment may vary markedly, particularly following meals. Accurately capturing the mean plasma enrichment with only a few measurements from a highly variable profile will thus be difficult. Finally, it is routinely assumed that plasma enrichment will rapidly drop to zero after the end of labeling. However, in both the one-day and seven-day labeling studies, subjects were not completely fasted (though diet was restricted) therefore glycogen will have been synthesized from D_2_-glucose during this period. Following the end of labeling, this store of “heavy glycogen” would be released, either as D_2_-glucose following glycogenolysis or as D_2_O following glycolysis in the liver. Consequently, label availability may be higher than expected following the withdrawal of intravenous D_2_-glucose. Errors caused by recycling of label back into the circulation from glycogen will be particularly problematic for short term labeling as the unaccounted for label will be a higher proportion of total label. Furthermore, because dietary carbohydrate was more tightly restricted in the seven-day labeling study, postprandial fluctuations and recycling would be reduced compared to the one-day labeling study. These factors may explain why a problem with the estimation of label availability is apparent in the one-day but not the seven-day D_2_-glucose labeling study. One advantage of long-term D_2_O labeling studies compared with short term D_2_-glucose labeling is that label can be normalized to a rapidly dividing cell population within the same individual. Consequently the level of label availability is internally controlled. This does however introduce two problems. One is that the normalization is usually performed using a cell type (typically granulocytes or monocytes) that is different from the cell population studied. Any differences in label availability or usage between the reference cell population and the studied population will introduce error. Secondly, the plateau enrichment in the reference population is estimated by modeling and may therefore be dependent on the choice of model. Why *b*_*w*_, which would be expected to be constant, varies so markedly between individuals remains unexplained and may hint at further problems with normalization in the case of D_2_O. Although the problems associated with normalization appear to be more severe in the case of D_2_-glucose, we do not advocate replacing D_2_-glucose labeling with D_2_O labeling as there are many objectives, including labeling of rapidly turning over populations, evaluating time-courses of appearance and disappearance of labeled cells, and labeling of cohorts of cells, that cannot be achieved with D_2_O. Instead, we recommend steps (outlined below) to reduce normalization errors.

Based on this work we recommend that, in the case of D_2_-glucose labeling, plasma glucose enrichment is measured at frequent intervals, during both day and night as well as following the withdrawal of label. It is important that sampling times are not all just before or just after meals (the former would lead to a systematic overestimate of label availability, the latter to a systematic underestimate). Tightly controlled dietary carbohydrate intake will also help to reduce fluctuations in plasma enrichment. Important directions for future work with D_2_-glucose include the development of approaches to estimate rapidly fluctuating plasma glucose enrichments (since continuous blood sampling is not acceptable) and experiments to more accurately estimate the true *in vivo* value of *b*_*g*_. In the case of longer term D_2_O labeling, which permits normalization to a rapidly turning over subpopulation, we recommend checking for model dependence in estimates of *b*_*w*_ and if necessary using the range of *b*_*w*_ to provide a range of estimated lymphocyte proliferation rates. Additionally, assessing the maximal enrichment from a range of reference cell populations will highlight whether there are between-cell type differences in label usage. Future directions for D_2_O include firstly, investigating why *b*_*w*_, which would be expected to be constant, varies between individuals and secondly investigating the impact of the choice of the reference cell population on normalization.

In summary, by combining *in vitro*, murine and human work with modeling we have demonstrated that biochemical differences between D_2_-glucose and D_2_O are not responsible for discrepancies in proliferation rates obtained with these methods. Instead we conclude that problems with normalizing the data play an important role. The problems are most acute for short term D_2_-glucose labeling where the rapid flux, diurnal variation and potential for label recycling make accurate estimation of plasma glucose levels difficult.

## Methods

### Reanalysis of existing T cell studies in humans: impact of length of labeling

To compare CD4^+^ T cell enrichment curves of different labeling studies, we calculated the enrichment level of total CD4^+^ T cells from a 9 week D_2_O labeling study [8], a seven-day D_2_-glucose labeling study [13] and a one-day D_2_-glucose labeling study in healthy individuals [6]. The seven-day D_2_-glucose labeling experiment directly studied total CD4^+^ T cells but the 9 week D_2_O experiment and the one-day D_2_-glucose experiments studied sorted “naïve” and “memory” CD4^+^ T cell subsets (defined as CD27^+^ CD45RO^-^ and CD45RO^+^ respectively in [8] and CD45RO^-^ and CD45RO^+^ respectively in [6]). For these two studies we used the enrichment data of naïve and memory CD4^+^ T cell subsets and their relative sizes within the total CD4^+^ T cell pool to recalculate the enrichment in total CD4^+^ T cells (total = naïve + memory). This approach could not be applied to total CD8^+^ T cells because there is a considerable fraction of CD27^-^CD45RO^-^ CD8^+^ effector T cells, meaning that combining CD27^+^CD45RO^-^ and CD45RO^+^ T cells is not equal to the total CD8^+^ population.

#### Multi-exponential model

To adjust for the length of the labeling period we fitted the data using a multi-exponential model [22,25]. The multi-exponential model assumes *N* subpopulations, the *i* ^th^ subpopulation has size *α*_*i*_, constant proliferation rate, *p*_*i*_ and constant disappearance rate, *d*_*i*_. The subpopulations are assumed to be independently at equilibrium and so *p*_*i*_ *= d*_*i*_. Fitting is repeated for increasing values of *N* (starting from *N* = 1); the value of *N* which optimizes the AICc is selected. The multi-exponential model takes the following form:

$$\frac{dA_{i}^{*}}{dt}=p_{i}bU(t)\alpha_{i}A-p_{i}A_{i}^{*}\;i=1\ldots N$$ (1)

Where *A* is the adenosine in the DNA of the total population, *A*_*i*_ * is the labeled adenosine in the DNA of sub-population *i*, *α*_*i*_ is the size of the i^th^ subpopulation (relative to the whole population), *p*_*i*_ is the proliferation rate of the i^th^ subpopulation, *b* the intracellular dilution or amplification factor (*b* = *b*_*w*_ for D_2_O, *b* = *b*_*g*_ for D_2_-glucose) and *U(t)* is the fraction of labeled precursors in the plasma at time *t*.

The multi-exponential model allows for saturation of label in rapidly turning over subpopulations but has a larger number of free parameters (2N-1) many of which are poorly identifiable, so it could not be used to estimate proliferation in the one-day D_2_-glucose labeling study where fewer data points were available. In the case of the one-day D_2_-glucose labeling study we therefore used the kinetic heterogeneity model.

#### Kinetic heterogeneity model

The kinetic heterogeneity model deals with heterogeneity implicitly by postulating that labeled cells may not be representative of the whole population and thus the disappearance rate of labeled cells (d*) may not be equal to the proliferation rate of the whole population (*p*) even for populations at equilibrium. The kinetic heterogeneity model takes the following form:

$$\frac{dA*}{dt}=pbU(t)A-d*A*$$ (2)

Where *A* is the total adenosine, A* is the labeled adenosine, *p* is the average proliferation rate of the whole population, d* the disappearance rate of labeled cells, *b* and *U(t)* as for the multi-exponential model.

The advantage of the kinetic heterogeneity model over the multi-exponential model is that it only has two free parameters both of which are identifiable and, in the absence of saturation, yields identical estimates of turnover of the whole population to the multi-exponential model (i.e. *p* estimated by the kinetic heterogeneity model equals $\sum_{i=1}^{N}\alpha_{i}p_{i}$ estimated by the multi-exponential model).

#### Model fitting

We find the parameters that minimize the difference between the experimental data and the modeled enrichment ($\sum_{i=1}^{N}\alpha_{i}A^{*}{}_{i}/A$ in the case of the multi-exponential model and *A* * */A* in the case of the kinetic heterogeneity model). Based on conventional normalisation techniques we fit *b*_*w*_ and fix *b*_*g*_ = 0.65. Fitting, here and throughout the project, was perfomed using the pseudorandom algorithm in the modFit function of the FME package in R [26,27].

### *In vitro* labeling

#### Stable isotope labeling

Jurkat cells were cultured in medium with either deuterated glucose (concentration 20%), deuterated water (concentration 2%), or both for seven-days and then for an additional seven-days in medium only. Medium was refreshed and cultures were split on regular intervals to prevent overcrowding in the culture. Cell numbers were determined using a hemocytometer and corrected for the dilution every time the media was replenished. DNA was sampled after 0 and 8h and subsequently on days 1, 2, 4, 5, 7, 8, 9, 11, 12 and 14 in most data sets. In four datasets timepoints 8h, 1d, 5d and 12d were replaced with measurements on day 3 and 10.

#### Measurement of deuterium enrichment in DNA

Enrichment of deuterium in DNA was assayed as previously described [1,19]. Briefly, DNA was extracted and hydrolyzed to deoxyribonucleotides and derivatized to penta-fluoro-triacetate. The deuterium content was analyzed by gas-chromatography/mass-spectrometry (GC/MS) using an Agilent 5973/6890 GC/MS system (Agilent Technologies). The derivative was injected into the GC/MS equipped with a HP-225 column and measured in SIM mode monitoring ions m/z 435 (M+0), and m/z 436 (M+1; for D_2_O labeling) or m/z 437 (M+2; for D_2_-glucose labeling). From the ratio of ions plasma deuterium enrichment was calculated by calibration against deoxyadenosine (for D_2_O labeling) or deoxyribose (for D_2_-glucose labeling) standards of known enrichment.

#### Modeling of in vitro data

We adjusted the previously published kinetic heterogenity models for D_2_-glucose [20] and D_2_O [8] labeling to account for a growing cell population; extension to the multi-exponential model did not change our conclusions. We defined a system of two ODEs, one for the unlabeled population (*R*) and one for the labeled population (*A* *):

$$\begin{array}{lll} \dot{R} & = & (1-bU)p(R+A^{*})-dR\\ {\dot{A}}^{*} & = & bUp(R+A^{*})-d^{*}A^{*} \end{array}\text{ during label administration}$$

$$\begin{array}{lll} \dot{R} & = & p(R+A^{*})-dR\\ {\dot{A}}^{*} & = & -d^{*}A^{*} \end{array}\text{ after label administration}$$

where *b* is the scaling factor for intracellular dilution (*b* = *b*_*g*_ for D_2_-glucose, *b* = *b*_*w*_ for D_2_O), *U* is the fraction of labeled precursor in the medium, *p* is the average proliferation rate of cells, *d* is the death rate of the unlabeled cells and *d* * is the death rate of the labeled cells. We assume death is negligible in Jurkat cultures without overcrowding i.e. *d* = *d* * = 0.

#### Model fitting

We found the parameters that minimized the difference between the modeled quantity *A* * */(A* * *+R)* and the experimental data. Based on conventional normalization techniques, we fitted *b*_*w*_ and fixed *b*_*g*_ = 0.65.

### *In vivo* labeling of mice

#### Ethics statement

Animal work was conducted at the St George's University of London (SGUL) Biological Research Facility, which is a designated establishment for animal research. The work in this study was approved by the SGUL Ethical Research Committee, as part of the process of obtaining the UK Home Office animal project licence (licence number: 70/7490). Due care was taken at all times to minimize suffering of animals during the experimentation. Experimental end-points always preceded the onset of any visible signs of suffering.

#### Stable isotope labeling

C57Bl/6J males, aged ~12 wk, were labeled with either deuterated (6,6-^2^H_2_-) glucose or deuterated water (D_2_O) for seven-days. For D_2_-glucose labeling, mice ate a 30% deuterated glucose liquid feed *ad libitum* instead of their normal chow for the labeling period of seven-days; to keep feeding circumstances similar, mice also received normal drinking water. For D_2_O labeling, mice were given an i.p. boost of 15ml/kg D_2_O (Cambridge Isotope Laboratories) in PBS at t = 0 and subsequently drank only 8% D_2_O; to keep circumstances similar, mice ate 8% D_2_O labeled liquid feed (Nutrison Standard 1.0 kcal/ml) *ad libitum* instead of their normal chow for the labeling period of seven-days following a run-in period of seven-days on a chemically-identical unlabeled liquid diet to ensure a stable metabolic status at the time of label introduction. For the duration of the experiment, weight of the mice, water consumption, and liquid feed consumption were monitored and recorded.

#### Sampling

Mice were sacrificed at 8 am on days 0, 1, 2, 3, 4, 7, 8, 10, 15, 21. Thymus and spleen were obtained by dissection and blood by cardiac puncture. Organs were mechanically disrupted to obtain single-cell suspensions. Blood was spun down to isolate plasma. Plasma was frozen and cells were cryopreserved in liquid nitrogen until further analysis. Prior to hydrolysis and derivatization for GC-MS analysis, samples with high cell numbers were processed using QiaGen DNA Mini Extraction kit to isolate DNA, and low-yield samples were boiled for an hour.

#### Measurement of deuterium enrichment in plasma and DNA

Deuterium enrichment in plasma and DNA from all sampling time points was analyzed by GC/MS. Plasma from D_2_-glucose labeled mice was derivatized to aldonitrile triacetate (ATA) as previously described. The derivative was injected into the GC/MS equipped with a HP-225 column (Agilent technologies) and measured in SIM mode monitoring ions m/z 328 (M+0) and m/z 330 (M+2). Plasma from D_2_O labeled mice was derivatized to acetylene (C_2_H_2_) as previously described. The derivative was injected into the GC/MS equipped with a PoraPLOT Q 25x0.32 column (Varian), and measured in SIM mode monitoring ions m/z 26 (M+0) and m/z 27 (M+1). From the ratio of ions plasma deuterium enrichment was calculated by calibration against standard glucose or water samples of known enrichment. DNA obtained from thymocytes, PBMC and splenocytes was analyzed as for *in vitro* labeling (see above).

### Modeling of deuterium enrichment in plasma and cell populations in mice

#### D_2_O

There are two normalization steps. A correction for the level of D_2_O in the plasma, *U(t)*, and a scaling factor, *b*_*w*_. Plasma enrichment was modeled by fitting a simple label enrichment/decay curve to the cross-sectional plasma enrichment data of all mice from the D_2_O labeling group:

$$\begin{array}{llll} U(t) & = & f(1-e^{-\delta t})+\beta e^{-\delta t} & \text{during labeling }t\leq \tau \\ U(t) & = & [f(1-e^{-\delta \tau })+\beta e^{-\delta \tau }]e^{-\delta (t-\tau )} & \text{after labeling }t>\tau \end{array}$$ (3)

as described previously [8], where *U(t)* represents the fraction of labeled precursor in plasma at time *t* (in days), *f* is the fraction of labeled precursor in the drinking water, *τ* is the length of the labeling period, *δ* is the turnover rate of body water per day, and *β* is the plasma enrichment attained after the boost of label by the end of day 0. We incorporated these best fits when analyzing the enrichment in different cell populations.

Up- and de labeling data of total thymocytes were analyzed as described previously [8], to estimate the maximum level of label intake that cells could possibly attain. The scaling factor *b*_*w*_ was then chosen such that this maximum level was 100%. Labeling data of PBMC and splenocytes were fitted with the kinetic heterogeneity model [20] (Eq (2)) and subsequently with a multi-exponential model [18,22] (Eq (1)).

#### D_2_-glucose

There are two normalization steps. A correction for the level of D_2_-glucose in the plasma, *U(t)*, and a normalization for intracellular dilution, *b*_*g*_. Since glucose is rapidly turned over rapid up and down labeling is readily achieved so, following the literature, we approximate the level of D_2_-glucose in the plasma with a square pulse.

$$\begin{array}{cccc} U(t) & = & U & t\leq \tau \\ U(t) & = & 0 & t>\tau \end{array}$$ (4)

Where U is equal to the average label enrichment in the plasma (calculated as the area under the enrichment-time curve). The intracellular normalization, *b*_*g*_, is set to 0.65 [2].

Labeling data of PBMC and splenocytes were fitted with the kinetic heterogeneity (Eq (2), [20]) and multi-exponential model (Eq (1), [22,25]).

### Impact of light-dark cycle on deuterium enrichment in plasma glucose

Six C57Bl/6J males, ~12weeks old, were housed in normal light/dark conditions; namely light for 12h from 08:00h to 20:00h and dark for 12h from 20:00h to 08:00h. A further 6 males were housed in reversed light conditions for 1 week prior to the experiment and then for the duration of the experiment (dark 08:00h-20:00, light 20:00–08:00h); this was to facilitate sampling of mice in scotophase during working hours.

All mice were labeled with deuterated (6,6-^2^H_2_-) glucose continuously. Mice ate a 30% deuterated glucose liquid feed *ad libitum* instead of their normal chow, mice also received normal drinking water.

### Modeling human monocyte data

Ethics statement: all human data were derived from existing studies. All data were anonymized and the original studies were approved by the relevant Ethics Review Boards.

We analyzed label enrichment in the DNA of monocytes from the one-day [6] and seven-day [13] D_2_-glucose studies. We constructed two new models, “bone marrow” and “delayed observation”:

### “Bone marrow” model

**Fig 8:**
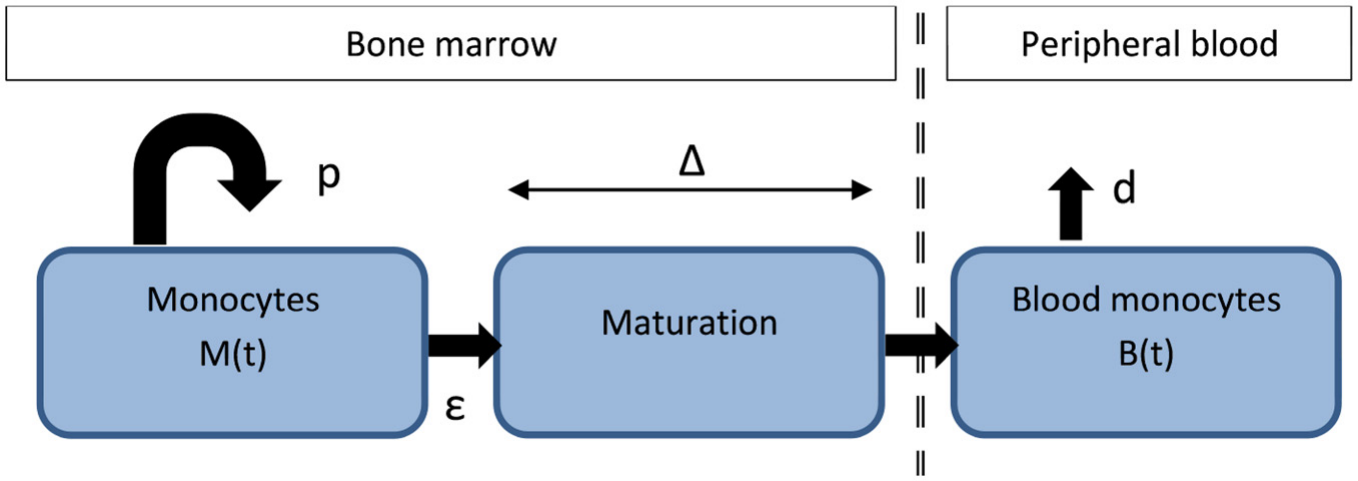
Model of monocyte development. Monocyte progenitors in the bone marrow (M(t)) proliferate at rate p, transit into a maturation compartment at rate ε, where they mature for a fixed time (Δ) before exiting into the blood compartment (B(t)), from which they disappear at rate d.

We constructed a model of monocyte development (Fig 8) in which monocyte progenitors in the bone marrow proliferate, mature for a fixed time, exit into the blood and from there disappear into tissue (to mature into macrophages).

In the absence of label the system is described by the following set of differential equations:

$$\begin{array}{lll} \dot{M}(t) & = & pM(t)-\varepsilon M(t)\\ \dot{B}(t) & = & \varepsilon M(t-\Delta )-dB(t) \end{array}$$ (5)

Where *M(t)* is the number of monocyte progenitors in the bone marrow at time t, *p* is their proliferation rate and *ε* their rate of exit into the maturation compartment prior to their exit into the blood. *B(t)* is the number of monocytes in peripheral blood, *Δ* is the time it take a monocyte progenitor to mature prior to entry into the blood and *d* is the rate of disappearance of blood monocytes from the blood into tissue. In the presence of label the system is described by

$$\begin{array}{llll} {\dot{A}}_{M}^{*} & = & \psi b_{g}pU(t)A_{M}-\varepsilon A_{M}^{*} & t\leq \tau \\ {\dot{A}}_{M}^{*} & = & -\varepsilon A_{M}^{*} & t>\tau \\ {\dot{A}}_{B}^{*} & = & \varepsilon A_{M}^{*}(t-\Delta )-dA_{B}^{*} & \forall t \end{array}$$ (6)

Where *A*_*M*_ and *A*_*M*_ * is total and labeled adenosine in DNA of monocytes in the bone marrow, *A*_*B*_ and *A*_*B*_ * is total and labeled adenosine in DNA of monocytes in the peripheral blood. *b*_g_ is the intracellular dilution, *ψ* is the maximal enrichment (will be equal to 1 if the normalization is working correctly), *τ* is the length of the labeling phase, *p*, *ε*, *Δ* and *d* as above. *U(t)* is the label enrichment in the plasma as defined in Eq(4).

We define *F*_*M*_ and *F*_*B*_ as the fractional label enrichment in marrow and blood monocytes respectively normalized by intracellular dilution *b*_g_ (fixed at 0.65) and plasma enrichment:

$$F_{M}(t)=\frac{A_{M}^{*}(t)}{b_{g}A_{M}U},\;F_{B}(t)=\frac{A_{B}^{*}(t)}{b_{g}A_{B}U}$$

Rearranging Eq (6) and eliminating *ε* and *A*_*M*_ */A*_*B*_ by assuming both blood and marrow monocytes are independently at equilibrium in Eq (5) gives:

$$\begin{array}{llll} {\dot{F}}_{M}^{} & = & \psi p-pF_{M}^{} & t\leq \tau \\ {\dot{F}}_{M}^{} & = & -pF_{M}^{} & t>\tau \\ {\dot{F}}_{B}^{} & = & dF_{M}^{}(t-\Delta )-dF_{B}^{} & \forall t \end{array}$$

This system was solved analytically and the theoretical quantity *F*_*B*_ fitted to the normalized monocyte data. Data from all individuals was pooled to avoid overfitting. We fitted *p*, *d*, *Δ* as population parameters (they each take a single value for the whole population) and *ψ* as an individual parameter (each subject has a different value of *ψ*). So, for example, in the seven-day D_2_-glucose study there are 3 subjects and hence 6 free parameters (*p*, *d*, *Δ*, *ψ*_*1*_, *ψ*_*2*,_ *ψ*_*3*_).

### “Delayed observation” model

We also considered a second model, the “delayed observation” model, which is a simple extension of the kinetic heterogeneity model which has been described previously [20]. Briefly, the delayed observation model (like the kinetic heterogeneity model) assumes a heterogeneous pool but deals with the heterogeneity implicitly [20] (to minimize free parameters). In the delayed observation model this pool represents the population of bone marrow monocytes and is therefore observed in the blood with a lag:

$$\begin{array}{llll} {\dot{F}}_{M}^{} & = & \psi p-d^{*}F_{M} & t\leq \tau \\ {\dot{F}}_{M}^{} & = & -d^{*}F_{M} & t>\tau \\ F_{B}(t) & = & F_{M}(t-\Delta )e^{-d*\Delta } & \forall t \end{array}$$

Where *F*_*M*_ is the fractional label enrichment in bone marrow monocytes, *p* is their proliferation rate, d* is the rate of disappearance of labeled monocytes, *ψ* is the maximal enrichment and *Δ* is the lag between an event in the pool and its observation in the blood (*F*_*B*_). The model was solved analytically and the theoretical quantity *F*_*B*_ *(t)* was fitted to the monocyte data normalized with the intracellular dilution and mean plasma enrichment. Data from all individuals was pooled. We fitted *p*, d*, *Δ* as population parameters and *ψ* as an individual parameter.

### Modeling human granulocyte data

We reanalyzed label enrichment in the DNA of granulocytes from the 9 week D_2_O study to estimate the scaling factor *b*_*w*_. We used the “bone marrow” model (described above) adjusted for D_2_O labeling:

$$\begin{array}{lll} {\dot{F}}_{M}^{} & = & b_{w}U(t)p-pF_{M}^{}\\ {\dot{F}}_{B}^{} & = & dF_{M}^{}(t-\Delta )-dF_{B}^{} \end{array}$$

Where *F*_*M*_ is the fractional label enrichment in bone marrow granulocytes, *p* is their proliferation rate, *d* is the rate of disappearance of labeled granulocytes, *U(t)* is an empirical function describing the level of enrichment in urine, *b*_*w*_ is the scaling factor and *Δ* is the lag between an event in the bone marrow and its observation in the blood (*F*_*B*_). For each of the five subjects studied (A-E) we used the previously estimated values of β, δ and f [8] to parameterize the function U(t) (Eq (3)). The theoretical quantity *F*_*B*_ *(t)* was fitted to the raw granulocyte data and the parameters *p*, *d*, *Δ* and *b*_*w*_ were estimated.

## Supporting Information

S1 Fig Normalized CD4^+^ T cell enrichment in one-day and seven-day D_2_-glucose labeling studies of humans. The percentage of labeled DNA in CD4^+^ T cells as observed in the seven-day (closed diamonds) and one-day (open circles) D_2_-glucose-labeling studies [6,13], **(A)** over the entire course of the experiment and **(B)** zoomed in to the first seven days. The end of the one-day and the seven-day labeling period are marked by the dashed gray and black lines, respectively. These data have been normalized for both the intracellular dilution (*b*_*g*_) and the availability of D_2_-glucose in the plasma. Therefore, everything being equal, we would expect the fraction of labeled DNA in CD4^+^ T cells at day 3 to be approximately 3 times higher in the seven-day labeling experiment than in the one-day labeling experiment (as individuals will have been labeled for 3 times longer and therefore 3 times as many cells will have divided). Instead the fraction of labeled cells is very similar in the two experiments.
(PDF)

S2 Fig Fit to *in vitro* labeling data. The model fits to the label enrichment in DNA from Jurkat cells cultured in medium with either D_2_-glucose, D_2_O or both for seven-days and then for an additional seven-days in medium only. Experiments 1–6 inclusive (first 12 graphs) were dual labeling experiments, so the cells in D_2_-glucose expt 1 are the same as the cells in D_2_O expt 1, etc. Expts 7 and above are independent datasets with no correspondence between the D_2_-glucose and the D_2_O expts. The proliferation rate estimates are plotted in Fig 2 and listed in S1 Table.
(PDF)

S3 Fig Plasma glucose and thymocyte DNA labeling in mice receiving D_2_-glucose. Deuterium labeling in plasma glucose taken both during light and dark phases (open circles, n = 27) and thymocyte DNA (filled diamonds, dotted line, n = 12) in 12 mice receiving oral feed labeled with D_2_-glucose.
(PDF)

S4 Fig Plasma glucose enrichment in humans. The deuterium enrichment in plasma glucose in 8 subjects from a one-day D_2_-glucose labeling study is shown [6]. 0h represents 8am, the start of the labeling period; the first measurement is taken immediately before the start of labeling. All individuals show considerable variations in enrichment over time and in all cases the enrichment increases over night (between 12h and 24h). The last time point (approx. 24h) was taken in the morning, immediately prior to breakfast; the one exception to this is C08 who had breakfast prior to the last measurement. Pearson correlations from 12h-24h: 0.99, 0.40, 0.56, 0.98, 0.97, 1.00, -0.25, 1.00. *P* = 0.03, two tailed Binary test. The increase in plasma enrichment during the night is in line with expectation as the infusion of labeled glucose continues whilst the ingestion of unlabeled glucose in food ceases; this also explains why C08 does not show an increase in plasma glucose enrichment at the last time point as, for this individual, the measurement was taken immediately after breakfast when unlabeled glucose would be high.
(PDF)

S5 Fig Fit of the “bone marrow” model to the fraction of DNA labeled in peripheral blood monocytes in healthy humans. Best fits (black lines) to deuterium enrichment in the DNA of peripheral blood monocytes. First row (subjects C2–C4) are subjects in the seven-day D_2_-glucose study [13]; rows 2–4 (subjects C02-C10) are subjects in the one-day D_2_-glucose study [6]. Prior *in vitro* experiments were used to determine the maximum percentage of labeled DNA that cells could possibly attain giving *b*_*g*_ = 0.65, (see Materials and Methods), and all measured enrichments were normalized by dividing by *b*_*g*_ *x area under the measured plasma glucose curve*. Each panel represents one subject, the end of label administration is marked by a dashed vertical line.
(PDF)

S6 Fig Fit of simple published model and bone marrow model to label in DNA of granulocytes in healthy humans. To normalize the label in DNA of T cells following a 9w D_2_O labeling protocol the maximum level of enrichment in granulocytes was estimated by fitting a simple mathematical model. Plots show the experimentally measured level of enrichment in DNA of granulocytes from 5 subjects **(A-E)** [8], the best fit of the simple, published model (red dotted line, [8]) and the best fit of the more physiological bone marrow model (solid blue line, see Materials and Methods). The lag *Δ* varies between individuals but the estimate of *b*_*w*_ is insensitive to this value.
(PDF)

S7 Fig Estimates of the intracellular dilution factor *b*_*w*_ in healthy humans. The intracellular dilution factor *b*_*w*_ is estimated by fitting a model to the level of enrichment in DNA of granulocytes. We considered two models, the simple published model [8] and a more physiological model (Materials & Methods). The fits of the models to the data are shown in S5 Fig, the resulting estimates of *b*_*w*_ are shown here. The 95% confidence intervals of the estimates are also plotted but are too small to be seen. The reanalysis results in a small but statistically significant decrease in *b*_*w*_. If T cell proliferation rates are estimated with this refined value of *b*_*w*_ then they increase significantly (*P* = 0.0003) but again the difference is numerically small (1–10%).
(PDF)

S1 Table Estimates of proliferation rate from *in vitro* D_2_-glucose and D_2_O labeling. Experiments 1–6 inclusive were dual labeling experiments, so the cells in D_2_-glucose experiment 1 are the same as the cells in D_2_O experiment 1, etc. Experiments 7 and above are independent datasets with no correspondence between the D_2_-glucose and the D_2_O experiments. Estimates are shown with 95% confidence intervals. These proliferation rate estimates are plotted in Fig 2, the experimental data and model fits are plotted in S2 Fig.
(PDF)
