# Supplementary material for: Reconciling Estimates of Cell Proliferation from Stable Isotope Labeling Experiments
Source: PLoS Comput Biol. 2015 Oct 5;11(10):e1004355. doi: 10.1371/journal.pcbi.1004355 (PMC4593553; doi:10.1371/journal.pcbi.1004355)
Supplement: S1 Fig — The percentage of labeled DNA in CD4+ T cells as observed in the seven-day (closed diamonds) and one-day (open circles) D2-glucose-labeling studies [6,13], (A) over the entire course of the experiment and (B) zoomed in to the first seven days. The end of the one-day and the seven-day labeling period are marked by the dashed gray and black lines, respectively. These data have been normalized for both the intracellular dilution (b g) and the availability of D2-glucose in the plasma. Therefore, everything being equal, we would expect the fraction of labeled DNA in CD4+ T cells at day 3 to be approximately 3 times higher in the seven-day labeling experiment than in the one-day labeling experiment (as individuals will have been labeled for 3 times longer and therefore 3 times as many cells will have divided). Instead the fraction of labeled cells is very similar in the two experiments. (PDF) [file pcbi.1004355.s001.pdf]

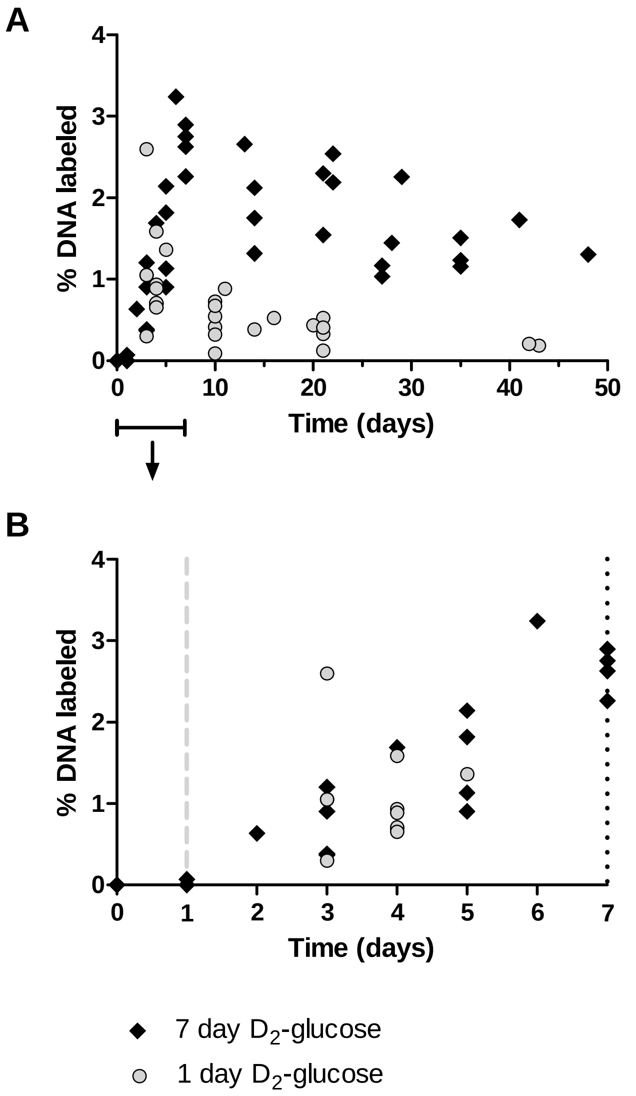

S1 Figure. Normalized CD4[+] T cell enrichment in one-day and seven-day D_2-glucose labeling studies of humans. The percentage of labeled DNA in CD4[+] T cells as observed in the seven-day (closed diamonds) and one-day (open circles) D_2-glucose-labeling studies [6,13], **(A)** over the entire course of the experiment and **(B)** zoomed in to the first seven days. The end of the one-day and the seven-day labeling period are marked by the dashed gray and black lines, respectively. This data has been normalized for both the intracellular dilution (b_g) and the availability of D_2-glucose in the plasma. Therefore, everything being equal, we would expect the fraction of labelled DNA in CD4+ T cells at day 3 to be approximately 3 times higher in the seven-day labelling experiment than in the one-day labelling experiment (as individuals will have been labelled for 3 times longer and therefore 3 times as many cells will have divided). Instead the fraction of labelled cells is very similar in the two experiments.
