# Supplementary material for: Reconciling Estimates of Cell Proliferation from Stable Isotope Labeling Experiments
Source: PLoS Comput Biol. 2015 Oct 5;11(10):e1004355. doi: 10.1371/journal.pcbi.1004355 (PMC4593553; doi:10.1371/journal.pcbi.1004355)
Supplement: S2 Fig — The model fits to the label enrichment in DNA from Jurkat cells cultured in medium with either D2-glucose, D2O or both for seven-days and then for an additional seven-days in medium only. Experiments 1–6 inclusive (first 12 graphs) were dual labeling experiments, so the cells in D2-glucose expt 1 are the same as the cells in D2O expt 1, etc. Expts 7 and above are independent datasets with no correspondence between the D2-glucose and the D2O expts. The proliferation rate estimates are plotted in Fig 2 and listed in S1 Table. (PDF) [file pcbi.1004355.s002.pdf]

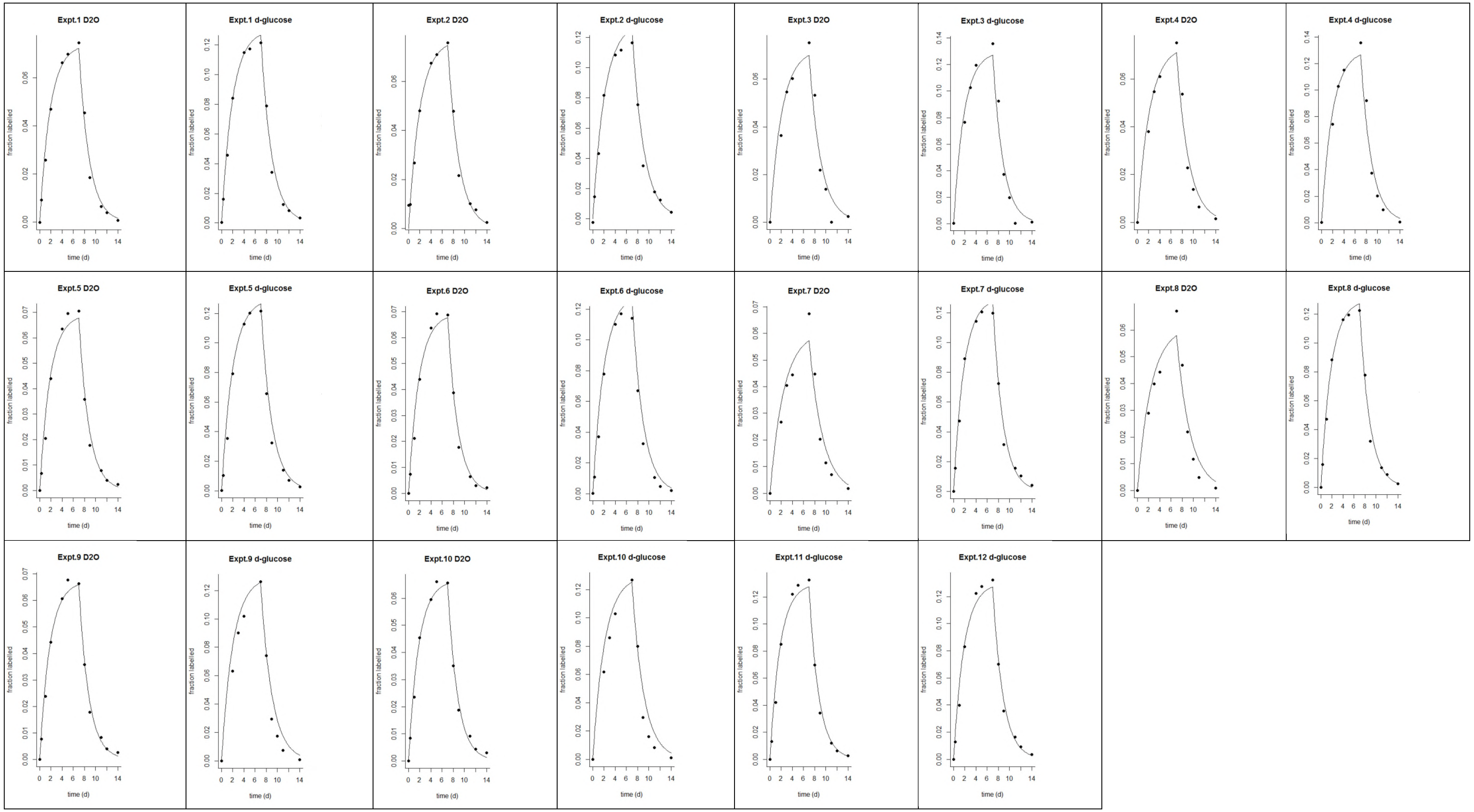

S2 Figure. Fit to *in vitro* labeling data. The model fits to the label enrichment in DNA from Jurkat cells cultured in medium with either D_2-glucose, D_2O or both for seven days and then for an additional seven days in medium only. Experiments 1-6 inclusive (first 12 graphs) were dual labeling experiments, so the cells in D_2-glucose expt 1 are the same as the cells in D_2O expt 1, etc. Expts 7 and above are independent datasets with no correspondence between the D_2-glucose and the D_2O expts. The proliferation rate estimates are plotted in Figure 2 and listed in S1 Table.
