# Supplementary material for: Reconciling Estimates of Cell Proliferation from Stable Isotope Labeling Experiments
Source: PLoS Comput Biol. 2015 Oct 5;11(10):e1004355. doi: 10.1371/journal.pcbi.1004355 (PMC4593553; doi:10.1371/journal.pcbi.1004355)
Supplement: S3 Fig — Deuterium labeling in plasma glucose taken both during light and dark phases (open circles, n = 27) and thymocyte DNA (filled diamonds, dotted line, n = 12) in 12 mice receiving oral feed labeled with D2-glucose. (PDF) [file pcbi.1004355.s003.pdf]

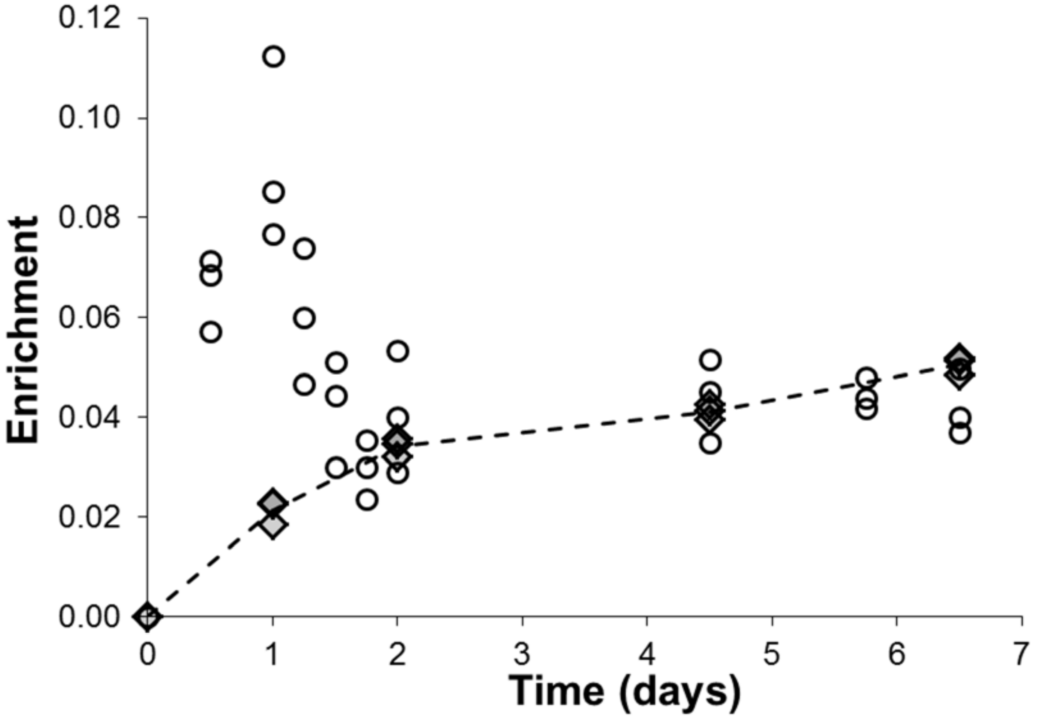

S3 Figure. Plasma glucose and Thymocyte DNA labeling in mice receiving D_2-glucose. Deuterium labeling in plasma glucose taken both during light and dark phases (open circles, n=27) and thymocyte DNA (filled diamonds, dotted line, n=12) in 12 mice receiving oral feed labeled with D_2-glucose.
