# Supplementary material for: Reconciling Estimates of Cell Proliferation from Stable Isotope Labeling Experiments
Source: PLoS Comput Biol. 2015 Oct 5;11(10):e1004355. doi: 10.1371/journal.pcbi.1004355 (PMC4593553; doi:10.1371/journal.pcbi.1004355)
Supplement: S4 Fig — The deuterium enrichment in plasma glucose in 8 subjects from a one-day D2-glucose labeling study is shown [6]. 0h represents 8am, the start of the labeling period; the first measurement is taken immediately before the start of labeling. All individuals show considerable variations in enrichment over time and in all cases the enrichment increases over night (between 12h and 24h). The last time point (approx. 24h) was taken in the morning, immediately prior to breakfast; the one exception to this is C08 who had breakfast prior to the last measurement. Pearson correlations from 12h-24h: 0.99, 0.40, 0.56, 0.98, 0.97, 1.00, -0.25, 1.00. P = 0.03, two tailed Binary test. The increase in plasma enrichment during the night is in line with expectation as the infusion of labeled glucose continues whilst the ingestion of unlabeled glucose in food ceases; this also explains why C08 does not show an increase in plasma glucose enrichment at the last time point as, for this individual, the measurement was taken immediately after breakfast when unlabeled glucose would be high. (PDF) [file pcbi.1004355.s004.pdf]

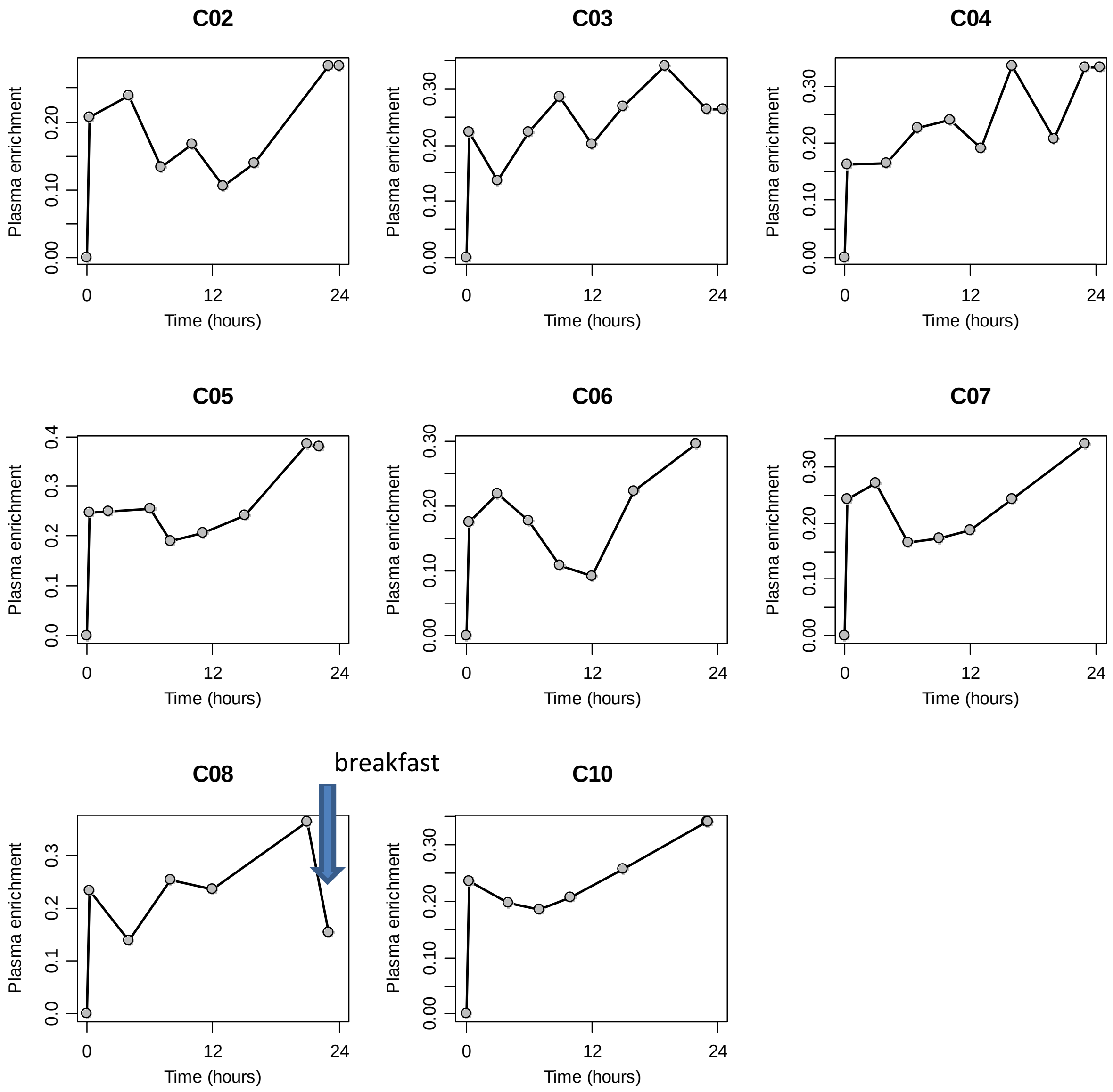

S4 Figure. Plasma glucose enrichment in humans. The deuterium enrichment in plasma glucose in 8 subjects from a one-day D_2-glucose labeling study is shown [6]. 0h represents 8am, the start of the labelling period; the first measurement is taken immediately before the start of labelling. All individuals show considerable variations in enrichment over time and in all cases the enrichment increases over night (between 12h and 24h). The last time point (approx. 24h) was taken in the morning, immediately prior to breakfast; the one exception to this is C08 who had breakfast prior to the last measurement. Pearson correlations from 12h-24h: 0.99, 0.40, 0.56, 0.98, 0.97, 1.00, -0.25, 1.00. *P*=0.03, two tailed Binary test. The increase in plasma enrichment during the night is in line with expectation as the infusion of labeled glucose continues whilst the ingestion of unlabeled glucose in food ceases; this also explains why C08 does not show an increase in plasma glucose enrichment at the last time point as, for this individual, the measurement was taken immediately after breakfast when unlabeled glucose would be high.
