# Supplementary material for: Reconciling Estimates of Cell Proliferation from Stable Isotope Labeling Experiments
Source: PLoS Comput Biol. 2015 Oct 5;11(10):e1004355. doi: 10.1371/journal.pcbi.1004355 (PMC4593553; doi:10.1371/journal.pcbi.1004355)
Supplement: S5 Fig — Best fits (black lines) to deuterium enrichment in the DNA of peripheral blood monocytes. First row (subjects C2–C4) are subjects in the seven-day D2-glucose study [13]; rows 2–4 (subjects C02-C10) are subjects in the one-day D2-glucose study [6]. Prior in vitro experiments were used to determine the maximum percentage of labeled DNA that cells could possibly attain giving b g = 0.65, (see Materials and Methods), and all measured enrichments were normalized by dividing by b g x area under the measured plasma glucose curve. Each panel represents one subject, the end of label administration is marked by a dashed vertical line. (PDF) [file pcbi.1004355.s005.pdf]

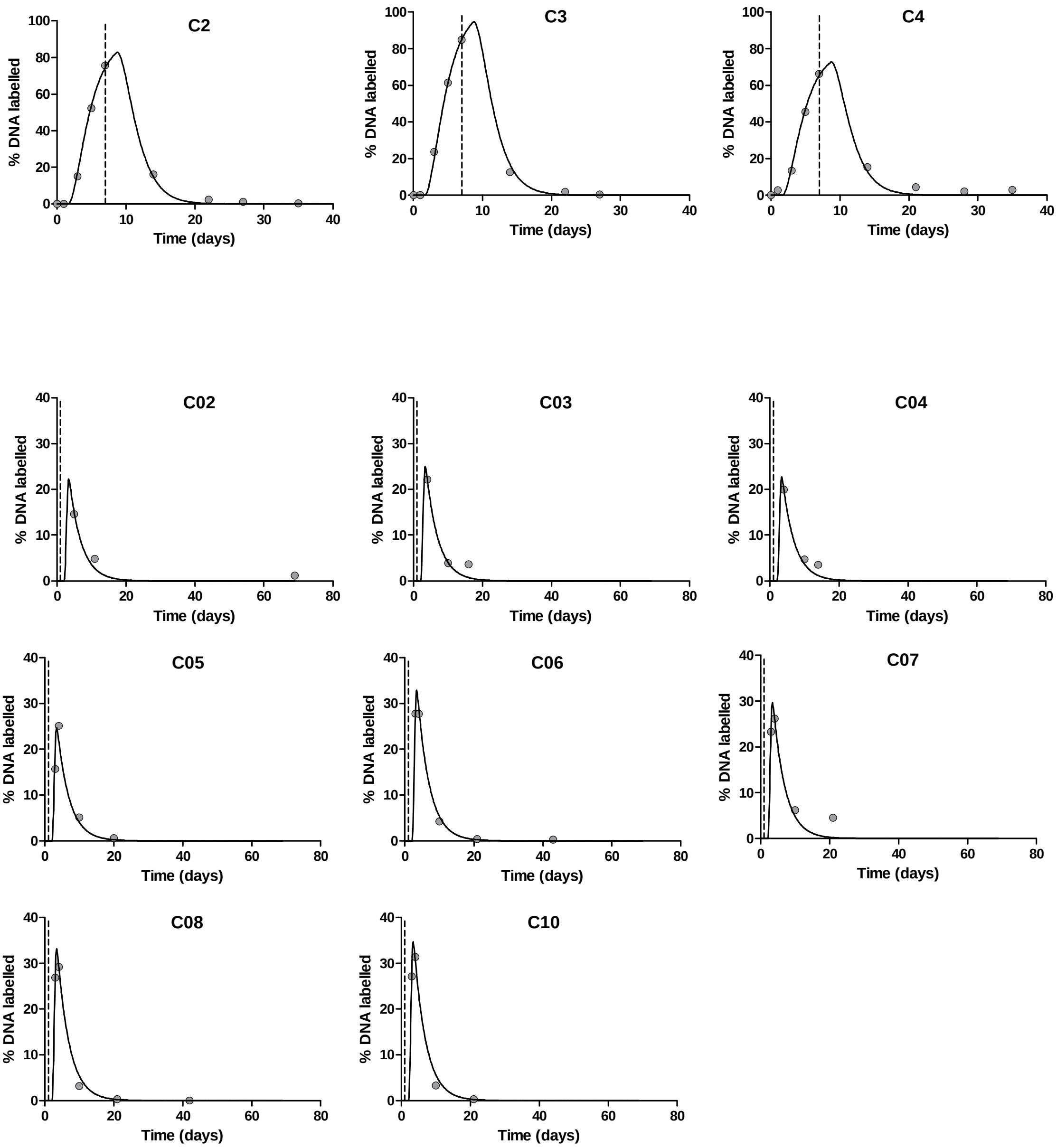

S5 Figure. Fit of the "bone marrow" model to the fraction of DNA labeled in peripheral blood monocytes in healthy humans. Best fits (black lines) to deuterium enrichment in the DNA of peripheral blood monocytes. First row (subjects C2-C4) are subjects in the seven-day D_2-glucose study [13]; rows 2-4 (subjects C02-C10) are subjects in the one-day D_2-glucose study [6]. Prior *in vitro* experiments were used to determine the maximum percentage of labeled DNA that cells could possibly attain giving b_g=0.65, (see Materials and Methods), and all measured enrichments were normalized by dividing by b_g *x area under the measured plasma glucose curve*. Each panel represents one subject, the end of label administration is marked by a dashed vertical line.
