# Supplementary material for: Reconciling Estimates of Cell Proliferation from Stable Isotope Labeling Experiments
Source: PLoS Comput Biol. 2015 Oct 5;11(10):e1004355. doi: 10.1371/journal.pcbi.1004355 (PMC4593553; doi:10.1371/journal.pcbi.1004355)
Supplement: S6 Fig — To normalize the label in DNA of T cells following a 9w D2O labeling protocol the maximum level of enrichment in granulocytes was estimated by fitting a simple mathematical model. Plots show the experimentally measured level of enrichment in DNA of granulocytes from 5 subjects (A-E) [8], the best fit of the simple, published model (red dotted line, [8]) and the best fit of the more physiological bone marrow model (solid blue line, see Materials and Methods). The lag Δ varies between individuals but the estimate of b w is insensitive to this value. (PDF) [file pcbi.1004355.s006.pdf]

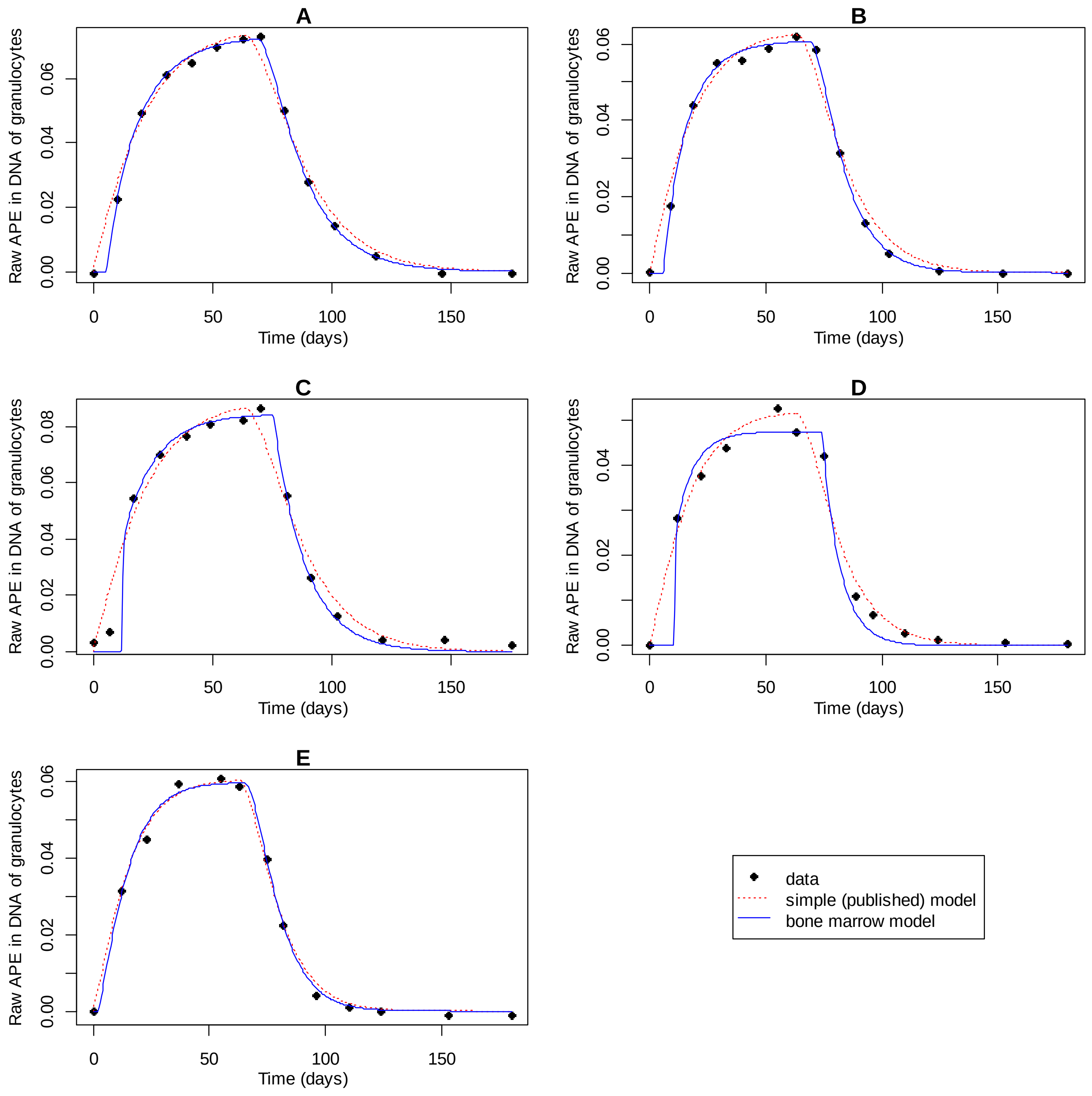

S6 Figure. Fit of simple published model and bone marrow model to label in DNA of granulocytes in healthy humans. To normalize the label in DNA of T cells following a 9w D_2O labeling protocol the maximum level of enrichment in granulocytes was estimated by fitting a simple mathematical model. Plots show the experimentally measured level of enrichment in DNA of granulocytes from 5 subjects (A-E) [8], the best fit of the simple, published model (red dotted line, [8]) and the best fit of the more physiological bone marrow model (solid blue line, see Materials and Methods). The lag Δ varies between individuals but the estimate of b_w is insensitive to this value.
