# Supplementary material for: Reconciling Estimates of Cell Proliferation from Stable Isotope Labeling Experiments
Source: PLoS Comput Biol. 2015 Oct 5;11(10):e1004355. doi: 10.1371/journal.pcbi.1004355 (PMC4593553; doi:10.1371/journal.pcbi.1004355)
Supplement: S7 Fig — The intracellular dilution factor b w is estimated by fitting a model to the level of enrichment in DNA of granulocytes. We considered two models, the simple published model [8] and a more physiological model (Materials & Methods). The fits of the models to the data are shown in S5 Fig, the resulting estimates of b w are shown here. The 95% confidence intervals of the estimates are also plotted but are too small to be seen. The reanalysis results in a small but statistically significant decrease in b w. If T cell proliferation rates are estimated with this refined value of b w then they increase significantly (P = 0.0003) but again the difference is numerically small (1–10%). (PDF) [file pcbi.1004355.s007.pdf]

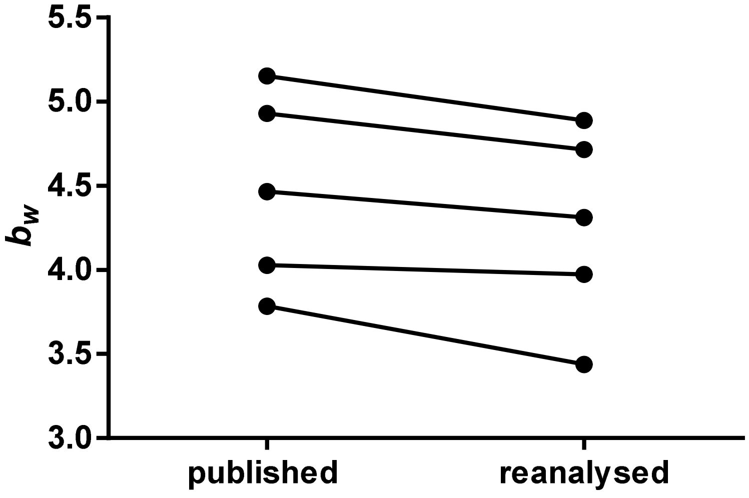

S7 Figure. Estimates of the intracellular dilution factor b_w in healthy humans. The intracellular dilution factor b_w is estimated by fitting a model to the level of enrichment in DNA of granulocytes. We considered two models, the simple published model [8] and a more physiological model (Materials & Methods). The fits of the models to the data are shown in S6 Figure, the resulting estimates of b_w are shown here. The 95% confidence intervals of the estimates are also plotted but are too small to be seen. The reanalysis results in a small but statistically significant decrease in b_w. If T cell proliferation rates are estimated with this refined value of b_w then they increase significantly (P=0.0003) but again the difference is numerically small (1-10%).
