# Supplementary material for: Reconciling Estimates of Cell Proliferation from Stable Isotope Labeling Experiments
Source: PLoS Comput Biol. 2015 Oct 5;11(10):e1004355. doi: 10.1371/journal.pcbi.1004355 (PMC4593553; doi:10.1371/journal.pcbi.1004355)
Supplement: S1 Table — Experiments 1–6 inclusive were dual labeling experiments, so the cells in D2-glucose experiment 1 are the same as the cells in D2O experiment 1, etc. Experiments 7 and above are independent datasets with no correspondence between the D2-glucose and the D2O experiments. Estimates are shown with 95% confidence intervals. These proliferation rate estimates are plotted in Fig 2, the experimental data and model fits are plotted in S2 Fig. (PDF) [file pcbi.1004355.s008.pdf]

S1 Table Estimates of proliferation rate from *in vitro* D_2-glucose and D_2O labeling.

		**Proliferation rate (day^{-1})**	
	**Expt.**	**D_2-glucose**	**D_2O**
matched	1	0.52 ± 0.05	0.54 ± 0.06
	2	0.48 ± 0.06	0.50 ± 0.07
	3	0.54 ± 0.11	0.48 ± 0.11
	4	0.52 ± 0.09	0.46 ± 0.08
	5	0.51 ± 0.09	0.55 ± 0.09
	6	0.50 ± 0.09	0.55 ± 0.08
unmatched	7	0.55 ± 0.06	0.41 ± 0.11
	8	0.55 ± 0.05	0.41 ± 0.11
	9	0.49 ± 0.11	0.56 ± 0.06
	10	0.47 ± 0.12	0.56 ± 0.06
	11	0.57 ± 0.07	
	12	0.54 ± 0.07	
**MEAN**		0.52	0.50
**STDEV**		0.03	0.06

Experiments 1-6 inclusive were dual labeling experiments, so the cells in D_2-glucose experiment 1 are the same as the cells in D_2O experiment 1, etc. Experiments 7 and above are independent datasets with no correspondence between the D_2-glucose and the D_2O experiments. Estimates are shown with 95% confidence intervals. These proliferation rate estimates are plotted in Figure 2, the experimental data and model fits are plotted in S2 Figure.
